# Genome-wide characterization and expression profiling of immune genes in the diamondback moth, *Plutella xylostella* (L.)

**DOI:** 10.1038/srep09877

**Published:** 2015-05-06

**Authors:** Xiaofeng Xia, Liying Yu, Minqian Xue, Xiaoqiang Yu, Liette Vasseur, Geoff M. Gurr, Simon W. Baxter, Hailan Lin, Junhan Lin, Minsheng You

**Affiliations:** 1 Institute of Applied Ecology, Fujian Agriculture and Forestry University, Fuzhou 350002, China; 2 Fujian-Taiwan Joint Centre for Ecological Control of Crop Pests, Fujian Agriculture and Forestry University, Fuzhou 350002, China; 3 Key Laboratory of Integrated Pest Management for Fujian-Taiwan Crops, Ministry of Agriculture, Fuzhou 350002, China; 4 School of biological sciences, University of Missouri-Kansas city, Kansas City, Missouri 64110-2499, USA; 5 Department of Biological Sciences, Brock University, 500 Glenridge Avenue, St. Catharines, Ontario, L2S 3A1 Canada; 6 Graham Centre, Charles Sturt University, Orange, New South Wales 2800, Australia; 7 School of Biological Sciences, the University of Adelaide, Adelaide, South Australia, Australia; 8 Fujian Vocational College of Bioengineering, Fuzhou 350002, China

## Abstract

The diamondback moth, *Plutella xylostella* (L.), is a destructive pest that attacks cruciferous crops worldwide. Immune responses are important for interactions between insects and pathogens and information on these underpins the development of strategies for biocontrol-based pest management. Little, however, is known about immune genes and their regulation patterns in *P. xylostella*. A total of 149 immune-related genes in 20 gene families were identified through comparison of *P. xylostella* genome with the genomes of other insects. Complete and conserved Toll, IMD and JAK-STAT signaling pathways were found in *P. xylostella*. Genes involved in pathogen recognition were expanded and more diversified than genes associated with intracellular signal transduction. Gene expression profiles showed that the IMD pathway may regulate expression of antimicrobial peptide (AMP) genes in the midgut, and be related to an observed down-regulation of AMPs in experimental lines of insecticide-resistant *P. xylostella*. A bacterial feeding study demonstrated that *P. xylostella* could activate different AMPs in response to bacterial infection. This study has established a framework of comprehensive expression profiles that highlight cues for immune regulation in a major pest. Our work provides a foundation for further studies on the functions of *P. xylostella* immune genes and mechanisms of innate immunity.

The innate immune system, which consists of cellular and humoral responses, is the first line of defense against bacterial and fungal infections in insects^1^. The humoral immune response includes the production of antimicrobial peptides (AMPs) that protect against a broad array of infectious agents, such as bacteria, fungi, viruses and even eukaryotic parasites^2^,^3^. In insects, the genetic mechanisms involved in these defence reactions have been most thoroughly studied in *Drosophila melanogaster*. In this model organism, genes encoding AMPs are activated by NF-κB transcription factors in response to infection through the Toll and immune deficiency (IMD) signaling pathways. Other immune pathways in insects are the JNK and JAK-STAT pathways, which participate in cell stress or wound response^4^,^5^,^6^. The JAK-STAT pathway also participates in antiviral response^7^. Different pattern recognition receptors (PRRs), such as peptidoglycan recognition proteins (PGRPs) and beta-1, 3-glucan recognition proteins (βGRPs), detect microbial infection and trigger the signaling cascades to activate the Toll and IMD pathways^1^.

The innate immune system is known to be highly conserved in the animal kingdom and evolutionarily very stable. It is, however, suggested that as insects are exposed to a multiplicity of continuously evolving pathogens, genes involved in signaling pathways may be under selection, leading to diversification^8^,^9^. Casanova-Torres and Goodrich-Blair^1^ compared the genetic mechanisms related to the immune system of *D. melanogaster* (Order: Diptera) and *Bombyx mori* and *Manduca sexta,* two representatives of Order: Lepidoptera. They showed, for example, that while some genetic components of the Toll signaling pathway may be similar between Lepidoptera and Diptera, some other genes differ. Gaps remain in some of these pathways and underline the need for further studies to better understand the differences between the Toll and IMD signalling pathways in these two insect orders. With greater availability of genetic tools and published genome sequences in Lepidoptera, it is possible to acquire a better understanding of the genetics of the immune system in other insect species such as *P. xylostella*, a notorious pest attacking many economically important food and oil crops in Brassicaceae.

Recent studies of *P. xylostella* immunity have mainly focused on individual immune genes and aspects such as production and characterization of cecropin^10^, the influence of endoparasitoids (*Cotesia vestalis*) on midgut proteinase activity, inhibition of phenoloxidase activity by metabolites of entomopathogenic nematodes (*Xenorhabdus nematophila*), and immune suppression by the parasitoid *Cotesia plutellae* polydnaviral gene^11^,^12^,^13^. Additionally, expressed sequence tags and cDNA microarrays have been used to analyze immune-inducible genes^14^. Whilst these studies of specific aspects are enlightening, there is a more fundamental need to develop an understanding of immune related genes based on genome-wide analysis, especially since the mechanisms of *P. xylostella* immunity at the molecular level are poorly understood.

Understanding the genetics and regulatory mechanisms of immune systems of insect pests, such as *P. xylostella*, has emerged as an important field to facilitate the development of effective biological control tactics. Comparative analyses of immune genes at the genome wide level in insects is the first step to define the candidate genes and functional networks associated with immune regulation. Thus, the aim of this study was to identify the immune signaling pathways, along with the pathogen recognition proteins and immune effectors, from the recently sequenced *P. xylostella* genome^15^. A total of 112 Serine proteases (SPs) and 102 serine protease homologs (SPHs) (9 SPs and 2 SPHs contain clip domains), as well as 26 serine protease inhibitors (Serpin) genes were also identified in the *P. xylostella* genome (data not shown), the detailed information on these components will be addressed in other paper. The expression patterns of immune genes that identified in this study were examined in *P. xylostella* at various development life stages, in different tissues and in susceptible and insecticide-resistant lines. Finally, *P. xylostella* immune response to bacterial pathogens was studied.

## Results

### Classification of *P. xylostella* immune genes

A total of 149 immune genes were identified in the *P. xylostella* genome, and classified into 20 families and 4 functional groups: immune recognition, signaling pathways, immune effectors, and others. The immune recognition genes included PGRPs, βGRPs, galectins, C-type lectins, fibrinogen-related proteins (FREPs), and scavenger receptors (SCRs). The signaling pathways genes were further classified into the pathway-related categories of TOLL, IMD, JNK and JAK-STAT. The immune effectors comprised prophenoloxidase (PPO), thioester-containing proteins (TEPs), and AMPs. Others included the enzyme superoxide dismutase (SOD), catalases and peroxidases that participate in detoxification of reactive oxygen species (ROS) (Table S1). The total number of *P. xylostella* immune genes was less than that of the other four insect species, *D. melanogaster*, *Anopheles gambiae*, *B. mori,* and *Tribolium castaneum*. The βGRPs were strikingly expanded, while AMPs were relatively reduced in *P. xylostella*.

### Immune recognition families

#### Peptidoglycan recognition proteins (PGRPs)

**Figure 1:**
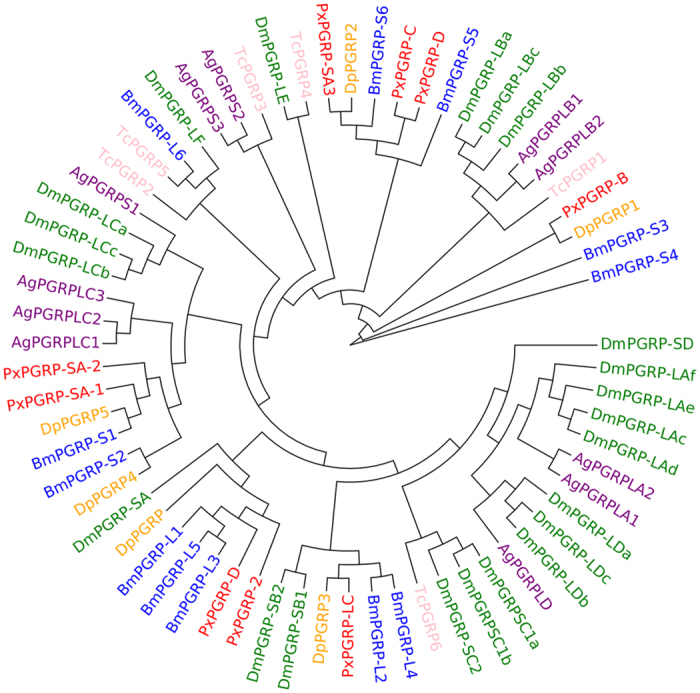
Phylogenetic analysis of PGRPs based on the sequences of *D. plexippus* (Dp), *B. mori* (Bm)*, P. xylostella* (Px), *D. melanogaster* (Dm), *A. gambiae* (Ag) and *T. castaneum* (Tc).

In this study, we identified 9 PGRP genes in *P. xylostella*, fewer than the 13 for *D. melanogaster*, 11 in *A. gambiae*, and 12 in *B. mori* (Table S1). Phylogenetic analysis showed that the 9 *P. xylostella* PGRPs were distributed in different branches and were homologous to *Danaus plexippus* and *B. mori* PGRPs, suggesting conservation of this protein family in Lepidoptera (Fig. 1). The 9 *P. xylostella* PGRPs were located in four different scaffolds (Table S2). Previous studies have shown that the Toll pathway is initiated by PGRP-SA^16^,^17^, thus the two PGRP-SAs (Px015207 and Px015209) in the same scaffold may function in the Toll pathway for the surveillance of Gram-positive bacteria. The IMD pathway, a conserved pathway that mainly defends against Gram-negative bacteria, is triggered through PGRP-LC^18^,^19^. *P. xylostella* PGRP-LC (Px004941) contains a trans-membrane region that may enable signal transmission into the cytoplasm and function in the IMD pathway. The domain architecture analysis (Fig. S1) suggests that all the putative *P. xylostella* PGRPs possess conserved domains, and Px001312 appears to be a secreted protein, whilst Px001311 possesses five transmembrane domains at the C-terminus, which may help signal transduction to the cytoplasm. Px008494 and Px004941 also exhibit possible amidase activity that can cleave the amide bond between N-acetylmuramoyl and L-amino acids in bacterial cell walls^20^.

#### Beta-1, 3-Glucan recognition proteins (βGRPs)

**Figure 2:**
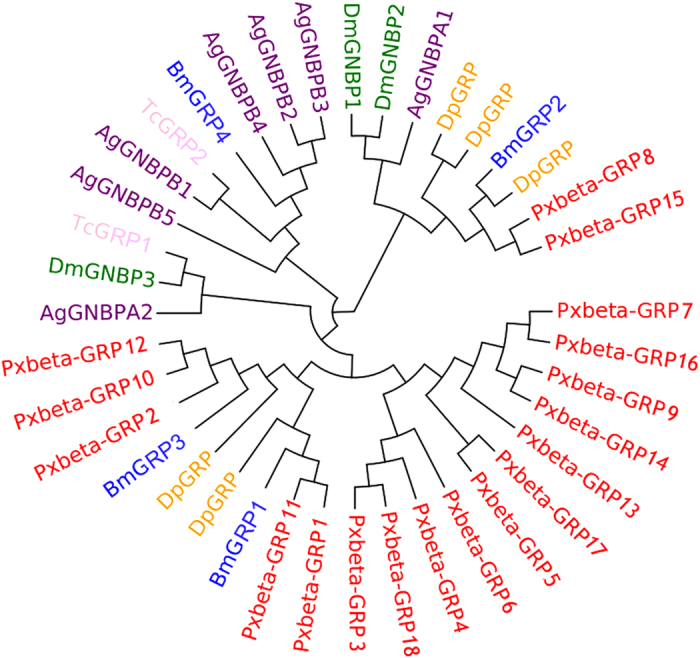
Phylogenetic analysis of GNBPs/βGRPs based on the sequences of *D. plexippus* (Dp), *B. mori* (Bm)*, P. xylostella* (Px), *D. melanogaster* (Dm), *A. gambiae* (Ag) and *T. castaneum* (Tc).

Although there are only 3 GNBPs in *D. melanogaster*, 7 in *A. gambiae*, and 4 βGRPs in *B. mori*, we found 18 βGRP genes in *P. xylostella*, indicating a dramatic expansion of this gene family in *P. xylostella* (Table S1). The 18 βGRPs of *P. xylostella* were clustered into three clades in the phylogenetic tree (Fig. 2), suggesting that the functions of this protein family might be diverse. Multiple sequence alignments and domain architecture analysis indicated that the conserved domains of glycosyl-hydrolase family 16 were present in *P. xylostella* βGRPs except for Px009706, which might have been an incomplete sequence or a pseudogene (Fig. S2). Previous studies indicated that the N-terminal regions of GNBPs/βGRPs also participate in carbohydrate recognition^21^,^22^. Our results showed that the N-terminal regions of *P. xylostella* βGRPs were more diverse than the C-terminal regions (Fig. S2), suggesting their importance in functional divergence. The 18 βGRPs were located in 7 scaffolds. The sequences with higher identities that looked like duplications in the phylogenetic tree, such as Px008677 (GRP7) and Px015064 (GRP16), Px001058 (GRP1) and Px009702 (GRP11), were not located in the same scaffolds (Table S2-3). The genes located in the same scaffold were those that were more diverse and distributed in different branches of the phylogenetic tree (Fig. 2), suggesting that there was no tandem duplication of βGRP genes in *P. xylostella* genome.

#### Galectins

Galectins are a family of lectins that contain evolutionary conserved carbohydrate-recognition domain (CRD) specifically for β-galactoside sugar^23^. Four galectins were identified in the *P. xylostella* genome (Table S2), and phylogenetic analysis showed that the *P. xylostella* galectins are homologous to those in other Lepidoptera insects such as *Danaus plexippus* and *B. mori* (Fig. S3). The domain architecture analysis (Fig. S4) suggests that all the *P. xylostella* putative galectins possess conserved CRD domains. Previous studies indicated that galectins function in innate immunity of *D. melanogaster* and *A. gambiae*, such as in microbial recognition and phagocytosis^24^. Therefore, the complete CRD domains in the sequences suggest a possible immune function in *P. xylostella*.

#### Fibrinogen-related proteins (FREPs)

FREPs are a family of proteins containing fibrinogen domains in the C-terminal region and they function in recognizing bacteria and parasites in invertebrates^25^,^26^. Two fibrinogen-related proteins were identified in the *P. xylostella* genome (Table S2). This family is large in *A. gambiae* with 61 genes in its genome, moderate in *D. melanogaster* with 14 genes and in *T. castaneum* with 7 genes, but quite small in the Lepidoptera (3 genes in *B. mori* and 2 genes in *P. xylostella* (Table S1). Phylogenetic analysis showed that the *P. xylostella* FREPs were most homologous to those of *B. mori*, and the genes in *D. melanogaster*, *A. gambiae*, and *T. castaneum* were more species-specific (Fig. S5). The domain architecture analysis (Fig. S6) indicated that all the putative *P. xylostella* FREPs possess conserved fibrinogen-related domains at the C-terminus, suggesting their possible full functions in the *P. xylostella* immunity.

#### C-type lectins

C-type lectins contain a wide variety of soluble and membrane-bound proteins with calcium-dependent carbohydrate-recognition domains (CRD)^27^. Previous studies argue that invertebrate C-type lectins play an important role in immune responses, such as activating PPO cascade^28^, participating in hemocyte nodule formation^29^, and recognizing microorganisms to enhance microbial clearance^30^. Seven C-type lectins were identified in the *P. xylostella* genome, which is much fewer than in *D. melanogaster*, *A. gambiae* and *B. mori* (Table S1). This might result from the incomplete information of the current *P. xylostella* genome^15^. It is also possible that this is a species-specific characteristic of *P. xylostella* since the βGRP family is expanded in *P. xylostella.* Phylogenetic analysis showed that the *P. xylostella* C-type lectins were homologous to those of *B. mori* and *D. plexippus*, and that there existed common 1:1 orthologs among the six species, indicating that the C-type lectins are conserved in insects (Fig. S7). The domain architecture analysis (Fig. S8) suggests that the putative *P. xylostella* C-type lectins possess one or two conserved carbohydrate-recognition domains which might function for the sugar-binding activity.

#### Scavenger receptors

The family of scavenger receptors was documented to contain multidomains and function as pattern recognition receptors in innate immunity^31^. This family can be divided into three subfamilies, scavenger receptors A (SCRAs), scavenger receptors B (SCRBs) and scavenger receptors C (SCRCs), based on their functional domains. The SCRAs have been implicated in the host defense by binding polyanionic ligands such as lipoteichoic acid (LTA) or lipopolysaccharide (LPS)^32^. The scavenger receptor Cysteine-Rich (SRCR) domain is usually located in some members of this subfamily, and contributes to binding to Gram-positive and Gram-negative bacteria^32^. One SCRA was identified in the *P. xylostella* genome (Table S2), and a SRCR domain is located at the middle of the sequence (Fig. S9), Phylogenetic analysis indicated the 1:1 ortholog between *P. xylostella* and *B. mori* (Fig. S10). SCRBs are thought to be a novel class of scavenger receptors characterized by a CD36 domain, they participated in the phagocytosis of microbes and binding to apoptotic cells^33^. Thirteen SCRBs were identified in the *P. xylostella* genome (Table S2), and they all contain a CD36 domain with one or two transmembrane domain(s) (Fig. S9). Phylogenetic analysis suggests that this subfamily in *P. xylostella* was more homologous to that in *D. plexippus* than in *B. mori* (Fig. S10). The SCRCs were previously identified to function as PRRs in phagocytosis and innate immunity^34^. This subfamily contained multidomains including two complement-control protein (CCP), one Meprin A5 antigen and RPTP Mu (MAM), and one somatomedin-B-like (BO). One SCRC was identified in the *P. xylostella* genome (Table S2) with 1 MAM, 1 BO, and a transmembrane domain in the sequence (Fig. S9). Phylogenetic analysis showed that this subfamily in *P. xylostella* was homologous to that in *D. plexippus* (Fig. S10).

### Immune signaling pathways

#### The Toll pathway

Spätzle (SPZ) is a ligand of the Toll receptor, and forms a complex with Toll receptor resulting in activation of the Toll signaling pathway^35^,^36^,^37^. There are six SPZ genes in *D. melanogaster* and *A. gambiae*. In *P. xylostella* genome, five SPZ genes were identified (Table S1). Common 1:1 orthologs or paralogs of SPZ genes were present in *P. xylostella*, *D. plexippus* and *B. mori* (Fig. S11), suggesting the conservative nature of this gene family in lepidopteran insects. Nine Toll receptor genes were identified in *P. xylostella* genome, with a gene duplication of Toll9. Phylogenetic analysis revealed gene duplication of Toll receptors in each of the five insect species (Fig. S12). The domain architecture analysis showed that six Toll receptors contain the functional domain of leucine rich repeats (LRRs), transmembrane domain (TM) and Toll/interleukin-1 receptor (IL-1R) homologous region (TIR) (Fig. S13), indicating that they may function in signal transduction. When Toll receptors are activated, they can bind to cytoplasmic MyD88 and, as a result, MyD88, tube and pelle form a complex to phosphorylate cactus, leading to degradation of cactus and release of Dorsal/Dif, which then translocate to the nucleus to activate antimicrobial peptide genes^16^. The Toll pathway genes of tube, pelle, cactus, TRAF6 and dorsal/dif but MyD88 were identified in *P. xylostella*. TRAF6 protein functions as an adapter in the shrimp *Litopenaeus vannamei* (Decapoda: Penaeidae) to regulate AMP gene expression^38^. However, function of *P. xylostella* TRAF6 needs to be further validated because *D. melanogaster* TRAF6 is not implicated in immune signaling^16^,^39^. The absence of MyD88 gene in *P. xylostella* might be due either to the homologue search method based on sequence similarity or incomplete genome information. It is also possible that the function of MyD88 is substituted by other adaptors. Further experiments would be needed to identify the cause.

#### The IMD pathway

The IMD pathway is a conserved pathway that is mainly activated by Gram-negative bacteria^16^,^36^. Genes in the IMD pathway, including IMD, Dredd, FADD, TAK1, TAB2, IKK-β and IKK-γ, were all identified in *P. xylostella* genome. Interestingly, TAK1 is not only a downstream component of IMD, but also a protein kinase that triggers the JNK pathway to activate gene expression in response to cell stress or wound^5^,^6^,^39^. JNK, C-Jun and Kay genes in the JNK pathway were all identified in *P. xylostella* genome. The common 1:1 orthologs of genes in the IMD and JNK pathways of *P. xylostella* and other insects indicated that these pathways were complete and conserved (Table S1-2). Three inhibitors of apoptosis 2 (IAP2) were also identified in *P. xylostella* genome (Table S1-2). IAP2 might be involved in Relish nuclear localization as evident in *Drosophila*^39^.

#### The JAK-STAT pathway

In *Drosophila*, only one cytokine receptor (Domeless), one Janus kinase (Hopscotch), and one transcription activator (STAT) have been identified in the JAK-STAT pathway^36^,^40^. However, one Domeless, one Hopscotch and two STATs homologues were identified in *P. xylostella* genome. In addition, only one TEP, which is under the control of the JAK-STAT pathway^4^, was identified in *P. xylostella* genome compared to other insect species (Table S1).

### Immune effectors

#### Antimicrobial peptides (AMPs)

AMPs are evolutionarily conserved proteins/peptides involved in innate immune responses. These peptides have small molecular weights and broad-spectrum of activities against bacteria, fungi and viruses^2^,^41^. Three AMP gene families, two cecropins, three moricins and two gloverins, were identified in *P. xylostella* genome. The seven AMP genes were located in 3 scaffolds, and AMPs of the same family were located in the same scaffold (Table S2). Cecropins are commonly found in insects, and are active against both Gram-positive and Gram-negative bacteria^42^. Phylogenetic analysis showed that *P. xylostella* cecropins were similar to those of *B. mori* and *D. plexippus*, and they formed a cluster in the tree (Fig. S14A). Moricin and gloverin have been found only in Lepidoptera thus far. Moricin acts against both Gram-positive and Gram-negative bacteria^43^. Although phylogenetic analysis showed that *P. xylostella* moricins were more homologous with *B. mori* moricins, different branches were formed in the tree and no 1:1 orthologs were found between the two moth species (Fig. S14B). Gloverin is a glycine-rich protein, which was first identified in *Hyalophora* pupae and was active against *E. coli*^44^. *B. mori* gloverins also exhibited activity against *E. coli* with rough lipopolysaccharide^45^, but studies of *M. sexta* gloverin revealed that it is active against both Gram-positive and Gram-negative bacteria, as well as fungi^46^. The gloverin family tended to be species-specific in the phylogenetic tree, and *P. xylostella* gloverins appeared to be more ancient in the tree (Fig. S14C). Lysozyme comprises a protein family that defends against bacteria by attacking peptidoglycans in cell walls, especially of Gram-positive bacteria^47^. Two lysozymes were identified in *P. xylostella* genome, and they are homologous to lysozymes in *D. plexippus* and *B. mori*.

#### Enzymes in reactive oxygen species (ROS) detoxification

Reactive oxygen species contribute to defense against invading microbial pathogens, but over-production can also harm host cells. Thus, ROS production must be tightly controlled. The concentration and conversion of ROS can be regulated by superoxide dismutases (SODs), peroxidases and catalases. SODs convert superoxide radical (O_2_^−^) into a less toxic product, hydrogen peroxide (H_2_O_2_). H_2_O_2_ is converted to water and oxygen by catalases. Peroxidase also scavenges H_2_O_2_ and converts it to hydroperoxide^48^,^49^,^50^. SODs are classified into two sub-families in *D. melanogaster*, with one family (Cu-Zn SOD) located in the cytosol and the other (Mn-Fe SOD) in the mitochondria. Seven SOD genes were identified in *P. xylostella* genome compared with 4 in *D. melanogaster*, 5 in *A. gambiae* and 6 in *B. mori*. Five of the 7 SODs in *P. xylostella* were Cu-Zn SOD, and the other two were Mn-Fe SODs, while three Cu-Zn SOD and one Mn-Fe SOD are present in *D. melanogaster*^36^. Orthologs were common in the SOD family (Fig. S15). Similarly, in the peroxidase family, orthologs were also common as shown in the phylogenetic tree (Fig. S16), but the catalase family was more species-specific (Fig. S17). The peroxidase family was expanded, with 17 peroxidase genes in *P. xylostella*, 20 in *D. melanogaster*, 26 in *A. gambiae* and 23 in *B. mori*. Thirteen catalase genes were identified in *P. xylostella*, significantly more than the 2 in *D. melanogaster*, 1 in *A. gambiae*, and 7 in *B. mori*. Previous studies have shown that plants attacked by insects may increase ROS production, resulting in oxidative damage to the insect midgut^51^. SODs, peroxidases and the expansion of catalases in *P. xylostella* genome is likely to reflect their roles in ROS detoxification and the wider co-evolution of this herbivore with cruciferous plants.

#### Prophenoloxidase (PPO)

PPO is an important enzyme for melanization in invertebrates to defend against pathogens and for wound healing^52^,^53^. PPO is cleaved by a serine protease cascade and converted to functional active enzyme phenoloxidase (PO). PO catalyzes the conversion of monophenols to quinones, thus contributing to melanin synthesis to defend against pathogens^52^,^53^. There are 3 PPO genes in *D. melanogaster*, 9 in *A. gambiae*, and 2 in *B. mori*. We identified only 1 PPO gene in the *P. xylostella* genome, which is highly supported by TblastN and manual NCBI blast annotation, and *P. xylostella* PPO formed a cluster with *B. mori* and *D. plexippus* PPOs in the phylogenetic tree (Fig. S18). The expansion of PPO is not common in insects, and there are only 2 PPOs in *M. sexta*^9^ and 3 in *T. castaneum*^54^. We also predicted another 8 PPO-like sequences with high TblastN scores; seven of them were located in the same scaffold. Manual annotation showed that these PPO-like sequences consist of three conserved domains, Hemocyanin_N, Hemocyanin_C and the copper-containing Hemocyanin_M. But NCBI blast results suggested that they were more homologous to hexamerin storage protein, which are hemocyanin-derived proteins with functions in amino acid storage^55^,^56^, juvenile hormone (JH) binding^57^ and in reproduction and metamorphosis^58^. Functional analysis needs to be performed to identify the actual roles of these proteins in *P. xylostella*.

### Expression analysis of immune genes in *P. xylostella*

#### Stage-specific expressions of immune genes

**Figure 3:**
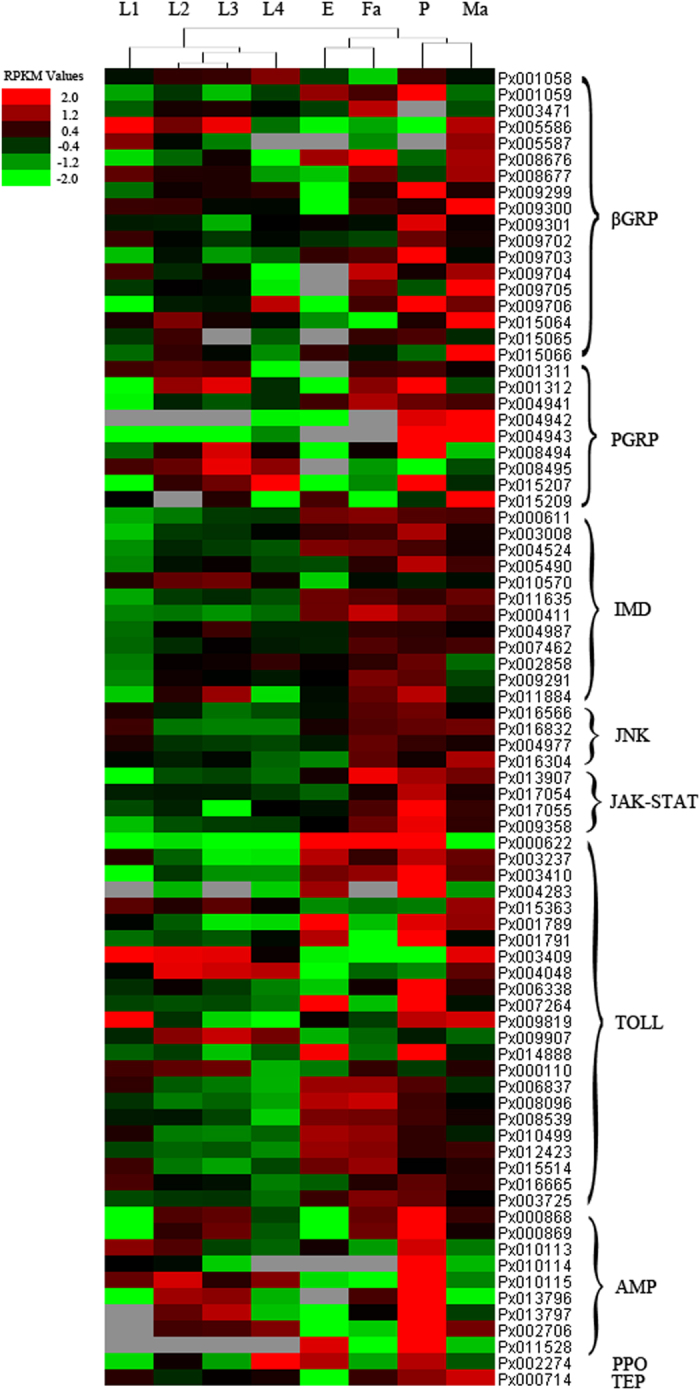
Expression profiling of immune genes at different developmental stages of *P. xylostella*. E: Egg; L1: 1^st^ instar larva; L2: 2^nd^ instar larva; L3: 3^rd^ instar larva; L4: 4^th^ instar larva; P: Pupa; Ma: Male adult; Fa: Female adult. Red represents the up-regulated genes, green indicates the down-regulated genes, and black represents no change in gene expression.

Expression profiles of immune genes were determined by RNA-seq^59^ from multiple life stages to show regulation patterns of different immune genes in *P. xylostella* (Fig. 3). PGRP and most βGRP genes were strongly expressed in pupae. The Toll pathway genes were highly expressed in eggs, 1^st^-instar larvae, pupae, and adults. The IMD pathway genes were highly expressed in pupae and adults whilst all AMP genes were highly expressed in pupae. The expression profiles of AMP genes at different developmental stages were also validated by qRT-PCR (Fig. S19). Our results indicated high expression levels of Toll and IMD pathways genes in pupae, resulting in high expression of AMPs. The JNK and JAK-STAT pathways genes were also highly expressed in pupae and adults, indicating their possible roles in cell stress or wound response.

#### Immune gene expression in the midgut and head

Much research has demonstrated that insect guts are related to immunity, such as the activation of host defense in the *Drosophila* gut in response to bacterial infection^60^ and the activation of AMPs by the IMD pathway in epithelia in response to infection^61^. Although the head is not generally considered an immune organ in insects, previous work has suggested that the honey bee head exhibits differential expression of proteins, including those that participate in signal transduction, in response to a bacterial challenge^62^. More generally, the head has many chemoreceptors, particularly associated with the mouthparts and antennae; is fundamental to feeding, the center for the nervous system and associated processing of various environmental signals including those associated with in mating^63^. Accordingly, the present study focused on the immune protection system in the *P. xylostella* head as well as the midgut to enrich the availability of information on insect immunity. Tissue-specific immune genes were differentially expressed in the heads of 4^th^-instar larvae, male and female adults, as well as in the midgut of 4^th^-instar larvae in *P. xylostella*. The results showed that four PGRP genes (Px008495, Px001312, Px008494 and Px004941) were strongly expressed in the 4^th^-instar larval midgut, while PGRP-SA (Px015207) was expressed at a higher level in the 4^th^-instar larval head. Most βGRP genes were highly expressed in the heads of both larvae and adults. In the midgut, only three βGRP genes (Px001058, Px001059 and Px009703) were strongly expressed (Fig. S20). Interestingly, our results indicated high expressions of βGRPs, SPZ, Toll receptor and Toll pathway genes in the adult head, but low expressions of these genes in the larval midgut. In contrast, the IMD pathway genes were all highly expressed in larval midgut, but expressed at lower levels in the larval head. Furthermore, AMPs of cecropin and gloverin were also highly expressed in the larval midgut. Previous studies have shown that the IMD pathway plays a critical role in activation of AMPs in epithelia in response to infection^61^. Therefore, we propose that, in *P. xylostella*, AMPs may be mainly regulated by the IMD pathway in the larval midgut, but by the Toll pathway in the larval head. The JNK and JAK-STAT pathways genes were highly expressed in the adult head but at lower expression levels in the larval midgut.

#### Strain-specific expressions of immune genes

AMP genes were all down-regulated in the chlorpyrifos and fipronil insecticide-resistant *P. xylostella* (CRL and FRL) compared to the susceptible strain (SS) (Fig. S21), which were confirmed by qRT-PCR (Fig. S22). Genes involved in the Toll pathway were affected by the insecticide resistance status of the *P. xylostella* strains; up-regulated in CRL but down-regulated in FRL compared to SS. However, the IMD pathway genes were down-regulated in both CRL and FRL, similar to the expression patterns of AMP genes. Thus, we hypothesize that repeated insecticide application may decrease expression of IMD pathway genes, resulting in down-regulation of AMP expression. A similar expression pattern was also found for lysozyme, which was down-regulated in the insecticide-resistant lines. In contrast to these down-regulated genes, those participating in the JNK and JAK-STAT pathways were all up-regulated in the resistant lines compared to the susceptible lines. Although it has been shown that the JNK pathway also regulates AMP production^64^, our finding of up-regulation of JNK pathway genes and down-regulation of AMPs suggest that the JNK pathway may not regulate AMP expression, at least in the insecticide-resistant lines. Previous studies showed that the JNK and JAK-STAT pathways participated in the cell stress or wound response^4^,^5^,^6^. The expression patterns of the JNK and JAK-STAT pathways genes suggest that the two pathways may respond to insecticide stress. Additionally, PPO genes were also up-regulated in the resistant lines, indicating their possible functions in response to insecticides (Fig. S21). Previous work indicated that *P. xylostella* phenoloxidase may play an important role in the increasing resistance to butane-fipronil^65^.

### Induced expression of AMPs by bacterial challenge

**Figure 4:**
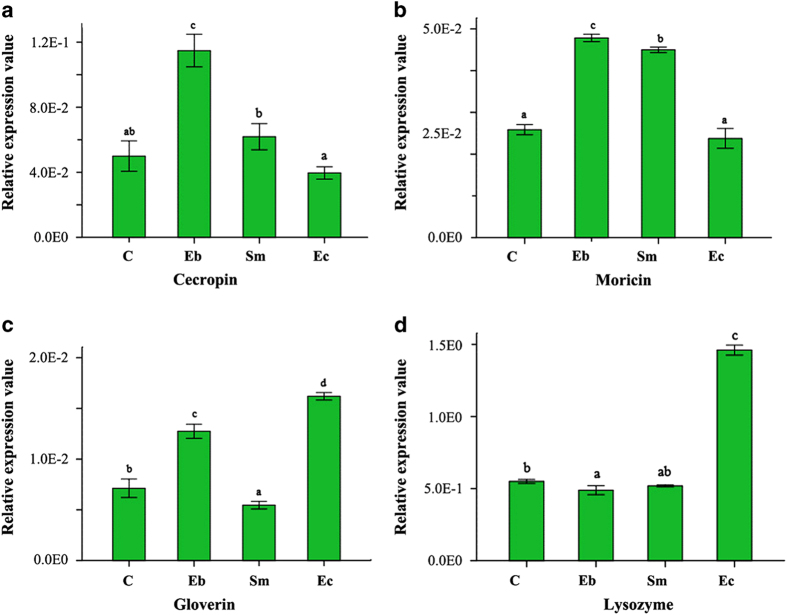
**Differential expressions of AMPs induced by bacterial infection.** C: Control, Eb: *Enterobacter* sp., Sm: *Serratia* sp., Ec: *Enterococcus* sp. Different letters over the columns within a graph denote significant differences (p < 0.05) among the different bacterial treatments, as determined by one-way ANOVA followed by LSD post hoc test.

Results from the bacteria-feeding experiment suggest that *P. xylostella* AMPs could be induced by bacterial infection (Fig. 4). After feeding on the Gram-negative bacterium *Enterobacter* sp., cecropin and moricin expressions were up-regulated significantly. Feeding *Serratia* sp. also significantly up-regulated moricin, but cecropins and moricins were not induced by the Gram-positive bacterium *Enterococcus* sp. Gloverin and lysozyme were up-regulated by *Enterococcus* sp., gloverin was also up-regulated by *Enterobacter* sp. These results indicate that *P. xylostella* may activate the expressions of specific immune effectors such as AMPs to defend against different types of pathogens. In *P. xylostella*, gloverin and lysozyme may mainly function in defense against Gram-positive bacteria, while cecropin and moricin may mainly defend against Gram-negative bacterial infection. Although Mackintosh *et al.*^66^ reported that gloverin isolated from the old world bollworm *Helicoverpa armigera* is active against Gram-negative bacteria (*Escherichia coli* and *Acinetobacter calcoaceticus*), but not active against Gram-positive bacteria (*Arthrobacter globiformis* and *Bacillus thuringiensis*), another study^46^ has reported that gloverin from *M. sexta* is active against Gram-positive bacteria (*Bacillus cereus*) but almost inactive against Gram-negative bacteria (*E. coli*). These studies suggest that gloverin in different insects may have species-specific functions in defense against pathogens.

## Discussion

**Figure 5:**
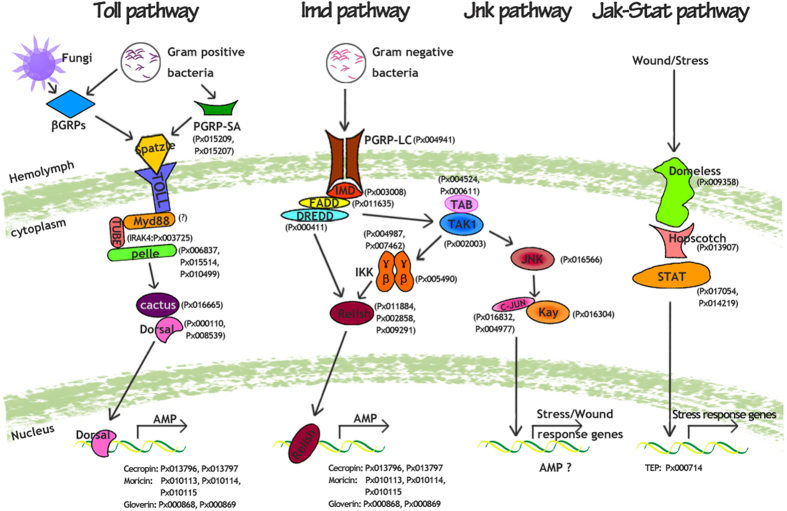
Proposed model of the potential immune signaling pathways in *P. xylostella* *P. xylostella* immune genes are indicated with prefix “Px” and are detailed in Table S2. All the putative pathways genes from *P. xylostella* were predicted based on sequence similarity as compared with other insects. The small question mark (?) next to the MyD88 indicates that the absence of this gene may be caused by the method of homologue search or incomplete genome information, and we cannot rule out the presence of MyD88 in *P. xylostella*. It is also possible that the function of MyD88 may be substituted by other adaptors, which need to be validated by experiments in future studies.

By comparative analysis of immune genes among five insect species, 20 gene families were identified in the *P. xylostella* genome, including components of the conserved immune signaling pathways (Toll, IMD, JNK and JAK-STAT), pathogen recognition and immune effectors. Based on the identified genes (Table S1-2) and their functions documented in other insect species^35^,^36^,^54^,^67^, we propose a model of the potential immune pathways in *P. xylostella* (Fig. 5) that need to be experimentally validated. The Toll and IMD pathways regulate synthesis of immune responsive effectors such as AMPs. The Toll pathway is also known to participate in *Drosophila* embryonic development^16^,^68^,^69^. Based on our current expression pattern of the Toll pathway genes, we believe that it might also play a similar role in *P. xylostella*. The JNK pathway is for transcriptional activation of defense genes or may participate in the synthesis of AMP, whilst the JAK-STAT pathway is for transcriptional activation of stress response genes like TEPs. As to the pathogen recognition gene families, we observed substantial expansion of βGRPs in *P. xylostella*, but also noted a large number of common 1:1 orthologs among the genes involved in intracellular signal transduction pathways related to immune responses. Previous studies have shown that *D. melanogaster* DmGNBP1 has a high binding affinity to lipopolysaccharide (LPS) and beta-1,3-glucan from bacteria and fungi, and can trigger the Toll pathway^70^. DmGNBP3 not only triggers the Toll pathway during fungal infection, but also activates the prophenoloxidase cascade^71^. In *M. sexta* larval plasma, β-1,3-glucanase-related protein may stimulate prophenoloxidase activation^72^,^73^. The large expansion of βGRPs in *P. xylostella* genome suggests that βGRPs may be involved in effective defense against Gram-negative bacteria and fungi. These results also suggest that while recognition proteins in the *P. xylostella* immune system may be diverse, the intracellular signal transduction genes may be more conserved. Previous studies in *D. melanogaster*^36^, *A. gambiae*^36^, and *B. mori*^35^ all suggested that the pathogen recognition receptors are more expanded and diverse than the genes participating in the intracellular signal transduction pathways. The expansion of diverse recognition receptors enables insects to recognize different pathogens and to trigger immune pathways that respond effectively to pathogens. This study also provides a foundation for future functional studies of βGRPs, which could be unique to *P. xylostella*. In contrast with βGRPs, only seven AMP genes in three families were identified in *P. xylostella* genome (Table S1-2). These results are consistent with those of previous studies using molecular cloning techniques^10^, EST and microarray to analyze immune-inducible genes^14^, or transcriptome analysis based on deep sequencing^74^. It remains unclear why there are fewer AMPs in *P. xylostella* than in other insects, such as *D. melanogaster*^36^*, A. gambiae*^36^ and *T. castaneum*^54^. Because our results were obtained from homologous search, we might have missed some AMP genes with sequences that are likely divergent from typical AMPs. It is also possible that reduction in common AMPs can be compensated by other unknown *P. xylostella*-specific AMP genes.

Gene expression profiles showed that most immune genes were highly expressed at the pupal stage and in the head tissue. Previous work showed that the bacterial pathogen *Campylobacter jejuni* could be transferred between life stages of *Musca domestica* (larva-pupa), but the number of *C. jejuni* declined during pupal development, coinciding with the increased expression of AMPs, indicating effective innate immunity at the pupal stage^75^. Thus, high expression of immune genes in the pupal stage of *P. xylostella* may be key to defense against pathogens and cell stress in this important developmental stage. On the other hand, since the head is the center for nervous system and feeding in insects with many important sense organs to perceive various environmental signals^63^, it might require a higher level of immune surveillance for adequate protection.

All the *P. xylostella* immune genes studied here were identified based on bioinformatics analysis. Their *in vivo* functions still need to be validated by molecular studies, such as gain-of-function or loss-of-function analysis. Nevertheless, through this study, we have generated information about the genetic composition and regulation of the immune system of an important agricultural pest. Our work will help drive future studies focusing on identifying molecular functions and mechanisms of immune genes and pathways of *P. xylostella* and other arthropods.

## Methods

### Identification and classification of immune genes in *P. xylostella*

The sequences of immune genes in *D. melanogaster* and *Anopheles gambiae* were downloaded from the immunology database at http://cegg.unige.ch/Insecta/immunodb. The *Bombyx mori* and *Tribolium castaneum* immune genes were edited in previous studies^35^,^54^, and were downloaded as queries. These immune-related sequences were used to search for immune genes in *P. xylostella* genome database (DBM-DB, http://iae.fafu.edu.cn/DBM/)^76^. A local TblastN search with an E-value of 10^−6^ was performed to collect putative immune genes from *P. xylostella* genome, and the default E-value of 1.0 was used to search for antimicrobial peptides (AMPs) that are 12-50 amino acids long and readily filtered out by high stringency conditions. As some AMPs, such as gloverin and moricin, could only be found in Lepidoptera, the relevant sequences from *D. plexippus*, *M. sexta* and *B. mori* were downloaded from the NCBI GenBank as queries to search for AMPs in *P. xylostella* database. The selected immune-related *P. xylostella* genes were then manually edited by comparing with *P. xylostella* transcriptome database ( http://iae.fafu.edu.cn/DBM/) and the Fgenesh program ( http://linux1.softberry.com/berry.phtml). Finally, the edited sequences were manually confirmed against the NCBI curated and conserved domains (CDD) database by blastX. Domain architecture was analyzed by the SMART ( http://smart.embl-heidelberg.de/), NCBI CDD database ( http://www.ncbi.nlm.nih.gov/Structure/cdd/docs/cdd_search.html) and PROSITE ( http://au.expasy.org/prosite/). Transmembrane domains were analyzed by TMHMM server v. 2.0 ( http://www.cbs.dtu.dk/services/TMHMM/). Signal peptide was analyzed by SignalP3.0 ( http://www.cbs.dtu.dk/services/SignalP/).

### Phylogenetic analysis

The phylogenetic relationships of immune genes were compared among six insect species: *P. xylostella*, *D. plexippus*, *B. mori*, *D. melanogaster*, *A. gambiae*, and *T. castaneum*. Multiple sequence alignments were performed with ClustalW and neighbor-joining method was used to construct phylogenetic trees by MEGA5.0 with 1000 bootstrap replicates.

### Expression patterns of immune genes in *P. xylostella*

RNA sequencing of different strains, developmental stages and tissues were performed previously in our laboratory^15^,^59^. Based on the *P. xylostella* transcriptome database (DBM-DB, http://iae.fafu.edu.cn/DBM/)^76^, expression profiles of immune genes were analyzed and clustered by Cluster3.0. The strain- (susceptible and insecticide resistant), stage- (E: Eggs; L1-L4: 1^st^, 2^nd^, 3^rd^ and 4^th^ instar larvae; P: Pupae; Ma: Male adults; Fa: Female adults) and tissue- (L4H: 4^th^ instar larval head; MaH: Male adult head; FaH: Female adult head; and L4Mg: 4^th^ instar larval midgut) specific patterns of expression were then visualized using Java TreeView.

### Insect rearing

A susceptible strain of *P. xylostella* (Fuzhou-S, hereafter abbreviated as SS) was collected in July 2004 from a vegetable field in Fuzhou (26.08 °N, 119.28 °E), Fujian province, southeastern China. All necessary permits were obtained from the Institute of Plant Protection at the Fujian Academy of Agricultural Sciences. Two insecticide resistant lines, chlorpyrifos- and fipronil-resistant lines (CRL and FRL), were selected previously^15^. The median lethal concentrations (LC_50_) for CRL and FRL were 574-fold (51,500.00 mg· L^−1^ vs. 89.79 mg L^−1^) and 72-fold (16.85 mg L^−1^ vs. 0.23 mg L^−1^) higher than the SS line, respectively. The three lines were reared on radish seedlings at 25 ± 2 °C, 70-80% RH and a 16 h light/8 h dark photoperiod without exposure to insecticides. Adults were fed with 10% honey solution and held in 500 mL plastic bottles for mating and oviposition. The newly hatched larvae were able to pass through holes on the bottom of bottles and drop onto radish plants underneath. Individuals at various developmental stages were collected for quantitative real-time PCR (qRT-PCR) analysis. The insects used for expression analysis of pathogen-induced immune gene were reared on radish seedlings to the third instar. The Gram-negative bacteria *Enterobacter* sp. (GenBank Accession Number: JQ396388) and *Serratia* sp. (JQ396393), and the Gram-positive bacterium *Enterococcus* sp. (KC150018) which were isolated from *P. xylostella* midgut by our lab were cultured in Luria Bertani (LB) media (10 g tryptone, 5 g yeast extract, 10 g NaCl, in one liter of distilled water, pH 7.0). The 3^rd^-instar larvae were placed in Petri dishes and starved for 12 hours. Cabbage leaves, dipped in a suspension of *Enterobacter* sp., *Serratia* sp. or *Enterococcus* sp. at OD_600_  =  1.0, were then added to the Petri dishes as diet. Cabbage leaves dipped in sterilized double distilled water were used as controls. *P. xylostella* larvae were allowed to feed on the cabbage leaves for 12 hours, and then collected for further analysis.

### Sample Collection for qRT-PCR

To investigate the changes in gene expression profiles during various life stages, newly laid *P. xylostella* eggs (~200), the 3^rd^-instar larvae, 4^th^-instar larvae, pupae, and adults (10 individuals for each of the developmental stages) were collected from the SS strain, samples were surface-sterilized with 75% ethanol for 60 sec, and then rinsed with DEPC water. The 3^rd^-instar larvae from CRL and FRL lines, as well as SS fed with the bacteria-dipped cabbage leaves were also collected (10 larvae per group). Total RNA was extracted from the whole bodies of each group using TRIzol (Takara Biotechnology (Dalian) Co., Ltd. (Takara Dalian)). cDNA was synthesized from 2 μg total RNA according to the instructions of GoScript^TM^ Reverse Transcription System (Promega, USA).

### qRT-PCR

qRT-PCR was performed to validate the expression profiles based on *P. xylostella* transcriptome, and evaluate immune response to bacterial infection. Primers for the tested genes are listed in Table S4. The ribosomal protein gene (RISC) was used as a control to calibrate the relative abundance of the representative genes. qRT-PCR contained 10 μL of GoTaq®qPCR Master Mix (Promega, USA), 7.2 μL of nuclease-free water, 2 μL of cDNA template from the representative samples (100 ng/μL final concentration), and 0.4 μL of each primer (10 mmol/L). qRT-PCR was performed in triplicate for each of three biological repeats in a BIO-RAD C1000 Touch^TM^ thermal cycler with cycling parameters as follows: initial denaturation at 95 °C for 3 min, followed by 35 cycles of 10 sec at 95 °C and 30 sec at 58 °C. To determine whether gene expressions in different lines, various stages or in response to bacterial infection were significantly different, data were analyzed by one-way ANOVA followed by LSD post hoc test using IBM SPSS Statistics 19.

## Author Contributions

X.X. and M.Y. designed the research; X.X., M.X., L.Y., H.L. and J.L. performed the research; X.X., M.Y., X.Y., S.B., G.M.G. and L.V. wrote and revised the manuscript.

## Additional Information

**How to cite this article**: Xia, X. *et al.* Genome-wide characterization and expression profiling of immune genes in the diamondback moth, Plutella xylostella (L.). *Sci. Rep.* **5**, 09877; doi: 10.1038/srep09877 (2015).

## Supplementary Material

Supplementary Information

## References

[b1] Casanova-Torres Á. M. & Goodrich-Blair H. Immune signaling and antimicrobial peptide expression in Lepidoptera. Insects 4, 320–338 (2013). 25861461 10.3390/insects4030320 PMC4386667

[b2] Bulet P. & Stocklin R. Insect antimicrobial peptides: structures, properties and gene regulation. Protein Pept. Lett. 12, 3–11 (2005). 15638797 10.2174/0929866053406011

[b3] Li Y., Xiang Q., Zhang Q., Huang Y. & Su Z. Overview on the recent study of antimicrobial peptides: origins, functions, relative mechanisms and application. Peptides 37, 207–215 (2012). 22800692 10.1016/j.peptides.2012.07.001

[b4] Agaisse H. & Perrimon N. The roles of JAK/STAT signaling in *Drosophila* immune responses. Immunol Rev. 198, 72–82 (2004). 15199955 10.1111/j.0105-2896.2004.0133.x

[b5] Botella J. A. *et al.* The *Drosophila* cell shape regulator c-Jun N-terminal kinase also functions as a stress-activated protein kinase. Insect Biochem. Molec. 31, 839–847 (2001). 10.1016/s0965-1748(01)00029-7 11439243

[b6] Rämet M., Lanot R., Zachary D. & Manfruelli P. JNK signaling pathway is required for efficient wound healing in Drosophila. Dev. Bio. 241, 145–156 (2002). 10.1006/dbio.2001.0502 11784101

[b7] Dostert C. *et al.* The JAK-STAT signaling pathway is required but not sufficient for the antiviral response of *Drosophila*. Nat. Immunol. 6, 946–953 (2005). 16086017 10.1038/ni1237

[b8] Lazzaro B. P. Natural selection on the *Drosophila* antimicrobial immune system. Curr. Opin. Microbiol. 11, 284–289 (2008). 18555739 10.1016/j.mib.2008.05.001 PMC2527063

[b9] Kanost M. R., Jiang H. & Yu X. Q. Innate immune responses of a lepidopteran insect, Manduca sexta. Immunol. Rev. 198, 97–105 (2004). 10.1111/j.0105-2896.2004.0121.x 15199957

[b10] Wang H. *et al.* Production, purification, and characterization of the cecropin from *Plutella xylostella*, pxCECA1, using an intein-induced self-cleavable system in *Escherichia coli*. Appl. Microbiol. Biot. 94, 1031–1039 (2012). 10.1007/s00253-011-3863-5 22258643

[b11] Shi M., Zhu N., Yi Y. & Chen X. X. Four serine protease cDNAs from the midgut of *Plutella xylostella* and theri proteinase activity are influenced by the endoparasitoid, *Cotesia vestalis*. Arch. Insect Biochem. 83, 101–114 (2013). 10.1002/arch.21097 23606528

[b12] Kim Y. G. & Song C. J. S. Bacterial metabolites of an entomopathogenic bacterium, *Xenorhabdus nematophila*, inhibit a catalytic activity of phenoloxidase of the diamondback moth, *Plutella xylostella*. J Microbiol. Biotechn. 21, 317–322 (2011). 21464604

[b13] Ali R. & Kim Y. A novel polydnaviral gene family, BEN, and its immunosuppressive function in larvae of *Plutella xylostella* parasitized by *Cotesia plutellae*. J Invertebr. Pathol. 110, 389–397 (2012). 22609480 10.1016/j.jip.2012.05.003

[b14] Eum J. H., Seo Y. R., Yoe S. M., Kang S. W. & Han S. S. Analysis of the immune-inducible genes of *Plutella xylostella* using expressed sequence tags and cDNA microarray. Dev. Comp. Immunol. 31, 1107–1120 (2007). 17379306 10.1016/j.dci.2007.02.002

[b15] You M. *et al.* A heterozygous moth genome provides insights into herbivory and detoxification. Nat. Genet. 45, 220–225 (2013). 23313953 10.1038/ng.2524

[b16] Valanne S., Wang J. H. & Rämet M. The *Drosophila* toll signaling pathway. J Immunol. 186, 649–656 (2011). 21209287 10.4049/jimmunol.1002302

[b17] Bischoff V. *et al.* Function of the *Drosophila* pattern-recognition receptor PGRP-SD in the detection of Gram-positive bacteria. Nat. Immunol. 5, 1175–1180 (2004). 15448690 10.1038/ni1123

[b18] Choe K. M., Werner T., Stöven S., Hultmark D. & Anderson K. V. Requirement for a peptidoglycan recognition protein (PGRP) in Relish activation and antibacterial immune responses in *Drosophila*. Science 296, 359–362 (2002). 11872802 10.1126/science.1070216

[b19] Gottar M. *et al.* The *Drosophila* immune response against Gram-negative bacteria is mediated by a peptidoglycan recognition protein. Nature 416, 640–644 (2002). 11912488 10.1038/nature734

[b20] Ward J. B., Curtis C. A., Taylor C. & Buxton R. S. Purification and characterization of two phage PBSX-induced lytic enzymes of *Bacillus subtilis* 168: an N-acetylmuramoyl-L-alanine amidase and an N-acetylmuramidase. J Gen. microbiol. 128, 1171–1178 (1982). 6126517 10.1099/00221287-128-6-1171

[b21] Hughes A. L. Evolution of the βGRP/GNBP/β-1, 3-glucanase family of insects. Immunogenetics 64, 549–558 (2012). 22410636 10.1007/s00251-012-0610-8

[b22] Mishima Y. *et al.* The N-terminal domain of *Drosophila* Gram-negative binding protein 3 (GNBP3) defines a novel family of fungal pattern recognition receptors. J Biol. Chem. 284, 28687–28697 (2009). 19692333 10.1074/jbc.M109.034587 PMC2781413

[b23] Pace K. E. *et al.* Characterization of a novel *Drosophila melanogaster* galectin. Expression in developing immune, neural, and muscle tissues. J Biol. Chem. 277, 13091–13098 (2002). 11809773 10.1074/jbc.M112105200

[b24] Pace K. E. & Baum L. G. Insect galectins: roles in immunity and development. Glycoconj J. 19, 607–614 (2004). 14758086 10.1023/B:GLYC.0000014092.86763.2f

[b25] Gokudan S. *et al.* Horseshoe crab acetyl group-recognizing lectins involved in innate immunity are structurally related to fibrinogen. Proc. Natl. Acad. Sci. USA 96, 10086–10091 (1999). 10468566 10.1073/pnas.96.18.10086 PMC17846

[b26] Adema C. M., Hertel L. A., Miller R. D. & Loker E. S. A family of fibrinogen-related proteins that precipitates parasite-derived molecules is produced by an invertebrate after infection. Proc. Natl. Acad. Sci. USA 94, 8691–8696 (1997). 9238039 10.1073/pnas.94.16.8691 PMC23082

[b27] Drickamer K. & Taylor M. E. Biology of animal lectins. Annu. Rev. Cell. Biol. 9, 237–264, (1993). 8280461 10.1146/annurev.cb.09.110193.001321

[b28] Yu X. Q. & Kanost M. R. Immulectin-2, a lipopolysaccharide-specific lectin from an insect, *Manduca sexta*, is induced in response to gram-negative bacteria. J Biol. Chem. 275, 37373–37381 (2000). 10954704 10.1074/jbc.M003021200

[b29] Koizumi N. *et al.* The lipopolysaccharide-binding protein participating in hemocyte nodule formation in the silkworm *Bombyx mori* is a novel member of the C-type lectin superfamily with two different tandem carbohydrate-recognition domains. FEBS Lett. 443, 139–143 (1999). 9989592 10.1016/s0014-5793(98)01701-3

[b30] Yu X. Q. & Kanost M. R. *Manduca sexta* lipopolysaccharide-specific immulectin-2 protects larvae from bacterial infection. Dev. Comp. Immunol. 27, 189–196 (2003). 12590970 10.1016/s0145-305x(02)00099-x

[b31] Philips J. A., Rubin E. J. & Perrimon N. *Drosophila* RNAi screen reveals CD36 family member required for *mycobacterial* infection. Science 309, 1251–1253 (2005). 16020694 10.1126/science.1116006

[b32] Peiser L., Mukhopadhyay S. & Gordon S. Scavenger receptors in innate immunity. Curr. Opin. Immunol 14, 123–128 (2002). 11790542 10.1016/s0952-7915(01)00307-7

[b33] Franc N. C., Heitzler P., Ezekowitz R. A. & White K. Requirement for croquemort in phagocytosis of apoptotic cells in *Drosophila*. Science 284, 1991–1994 (1999). 10373118 10.1126/science.284.5422.1991

[b34] Ramet M. *et al.* *Drosophila* scavenger receptor CI is a pattern recognition receptor for bacteria. Immunity 15, 1027–1038 (2001). 11754822 10.1016/s1074-7613(01)00249-7

[b35] Tanaka H. *et al.* A genome-wide analysis of genes and gene families involved in innate immunity of *Bombyx mori*. Insect Biochem. Molec. 38, 1087–1110 (2008). 10.1016/j.ibmb.2008.09.001 18835443

[b36] Waterhouse R. M. *et al.* Evolutionary dynamics of immune-related genes and pathways in disease-vector mosquitoes. Science 316, 1738 (2007). 17588928 10.1126/science.1139862 PMC2042107

[b37] Hu X., Yagi Y., Tanji T., Zhou S. & Ip Y. T. Multimerization and interaction of Toll and Spätzle in *Drosophila*. P. Natl. Acad. Sci. USA 101, 9369–9374 (2004). 10.1073/pnas.0307062101 PMC438983 15197269

[b38] Wang P. H. *et al.* *Litopenaeus vannamei* tumor necrosis factor receptor-associated factor 6 (TRAF6) responds to *Vibrio alginolyticus* and white spot syndrome virus (WSSV) infection and activates antimicrobial peptide genes. Dev. Comp. Immunol. 35, 105–114 (2011). 20816892 10.1016/j.dci.2010.08.013

[b39] Kleino A. *et al.* Inhibitor of apoptosis 2 and TAK1-binding protein are components of the *Drosophila* Imd pathway. EMBO J. 24, 3423–3434 (2005). 16163390 10.1038/sj.emboj.7600807 PMC1276168

[b40] Harrison D. A. The JAK/STAT pathway. Csh. Perspect. Biol. 4, a011205; 10.1101/cshperspect.a011205 (2012).

[b41] Bulet P., Stöcklin R. & Menin L. Anti-microbial peptides: from invertebrates to vertebrates. Immunol. Rev. 198, 169–184 (2004). 15199962 10.1111/j.0105-2896.2004.0124.x

[b42] Samakovlis C., Kimbrell D. A., Kylsten P., Engström Å. & Hultmark D. The immune response in *Drosophila*: pattern of cecropin expression and biological activity. EMBO J. 9, 2969–2976 (1990). 2390977 10.1002/j.1460-2075.1990.tb07489.x PMC552014

[b43] Hara S. & Yamakawa M. Moricin, a novel type of antibacterial peptide isolated from the silkworm, *Bombyx mori*. J Biol. Chem. 270, 29923–29927 (1995). 8530391 10.1074/jbc.270.50.29923

[b44] Axén A., Carlsson A., Engström Å. & Bennich H. Gloverin, an antibacterial protein from the immune hemolymph of *Hyalophora* pupae. Eur. J. Biochem. 247, 614–619 (1997). 9266704 10.1111/j.1432-1033.1997.00614.x

[b45] Yi H. Y. *et al.* Gloverins of the silkworm *Bombyx mori*: structural and binding properties and activities. Insect Biochem. Molec. 43, 612–625 (2013). 10.1016/j.ibmb.2013.03.013 PMC3760519 23567591

[b46] Xu X. X., Zhong X., Yi H. Y. & Yu X. Q. *Manduca sexta* Gloverin binds microbial components and is active against bacteria and fungi. Dev. Comp. Immunol. 38, 275–284 (2012). 22858411 10.1016/j.dci.2012.06.012 PMC3443299

[b47] HA M. & FH W. Lysozyme and alpha-lactalbumin: structure, function, and interrelationships. Adv. Protein Chem. 41, 173–315 (1991). 2069076 10.1016/s0065-3233(08)60198-9

[b48] DeJong R. J. *et al.* Reactive oxygen species detoxification by catalase is a major determinant of fecundity in the mosquito *Anopheles gambiae*. P. Natl. Acad. Sci. USA 104, 2121–2126 (2007). 10.1073/pnas.0608407104 PMC1892935 17284604

[b49] Mittapalli O., Neal J. J. & Shukle R. H. Antioxidant defense response in a galling insect. P. Natl. Acad. Sci. USA 104, 1889–1894 (2007). 10.1073/pnas.0604722104 PMC1783901 17261812

[b50] Bandyopadhyay U., Das D. & Banerjee R. K. Reactive oxygen species: oxidative damage and pathogenesis. Curr. Sci. India 77, 658–666 (1999).

[b51] Bi J. & Felton G. Foliar oxidative stress and insect herbivory: primary compounds, secondary metabolites, and reactive oxygen species as components of induced resistance. J Chem. Ecol. 21, 1511–1530 (1995). 24233680 10.1007/BF02035149

[b52] Li Y., Wang Y., Jiang H. & Deng J. Crystal structure of *Manduca sexta* prophenoloxidase provides insights into the mechanism of type 3 copper enzymes. P. Natl. Acad. Sci. USA 106, 17002–17006 (2009). 10.1073/pnas.0906095106 PMC2761362 19805072

[b53] Cerenius L., Lee B. L. & Söderhäll K. The proPO-system: pros and cons for its role in invertebrate immunity. Trends Immunol. 29, 263–271 (2008). 18457993 10.1016/j.it.2008.02.009

[b54] Zou Z. *et al.* Comparative genomic analysis of the *Tribolium* immune system. Genome Biol. 8, R177 (2007). 17727709 10.1186/gb-2007-8-8-r177 PMC2375007

[b55] Burmester T. & Scheller K. Ligands and receptors: common theme in insect storage protein transport. Naturwissenschaften 86, 468–474 (1999). 10541655 10.1007/s001140050656

[b56] Martins J., Nunes F., Cristino A., Simões Z. & Bitondi M. The four hexamerin genes in the honey bee: structure, molecular evolution and function deduced from expression patterns in queens, workers and drones. BMC Mol. Biol. 11, 23 (2010). 20346164 10.1186/1471-2199-11-23 PMC2861669

[b57] Ismail S. M. & Gillott C. Identification, characterization, and developmental profile of a high molecular weight, juvenile hormone‐binding protein in the hemolymph of the migratory grasshopper, *Melanoplus sanguinipes*. Arch. Insect Biochem. 29, 415–430 (1995).

[b58] Pan M. & Telfer W. H. Methionine-rich hexamerin and arylphorin as precursor reservoirs for reproduction and metamorphosis in female luna moths. Arch. Insect Biochem. 33, 149–162 (1996). 10.1002/(SICI)1520-6327(1996)33:2<149::AID-ARCH5>3.0.CO;2-T 8864211

[b59] He W. *et al.* Developmental and insecticide-resistant insights from the de novo assembled transcriptome of the diamondback moth, *Plutella xylostella*. Genomics 99, 169–177 (2012). 22240003 10.1016/j.ygeno.2011.12.009

[b60] Buchon N., Broderick N. A., Poidevin M., Pradervand S. & Lemaitre B. *Drosophila* intestinal response to bacterial infection: activation of host defense and stem cell proliferation. Cell Host Microbe. 5, 200–211 (2009). 19218090 10.1016/j.chom.2009.01.003

[b61] Tzou P. *et al.* Tissue-specific inducible expression of antimicrobial peptide genes in *Drosophila* surface epithelia. Immunity 13, 737–748 (2000). 11114385 10.1016/s1074-7613(00)00072-8

[b62] Scharlaken B. *et al.* Differential protein expression in the honey bee head after a bacterial challenge. Arch. Insect Biochem. Physiol. 65, 223–237 (2007). 17630657 10.1002/arch.20179

[b63] Wessnitzer J. & Webb B. Multimodal sensory integration in insects-towards insect brain control architectures. Bioinspir. Biomim. 1, 63–75 (2006). 17671308 10.1088/1748-3182/1/3/001

[b64] Wojda I., Kowalski P. & Jakubowicz T. JNK MAP kinase is involved in the humoral immune response of the greater wax moth larvae *Galleria mellonella*. Arch. Insect. Biochem. 56, 143–154 (2004). 10.1002/arch.20001 15274175

[b65] Liu S. *et al.* Does phenoloxidase contributed to the resistance? selection with butane-fipronil enhanced its activities from diamondback moths. Open Biochem. J. 3, 9–13 (2009). 19401784 10.2174/1874091X00903010009 PMC2674291

[b66] Mackintosh J. A. *et al.* A gloverin-like antibacterial protein is synthesized in *Helicoverpa armigera* following bacterial challenge. Dev Comp. Immunol. 22, 387–399 (1998). 9699484 10.1016/s0145-305x(98)00025-1

[b67] Evans J. *et al.* Immune pathways and defence mechanisms in honey bees *Apis mellifera*. Insect. Mol. Biol. 15, 645–656 (2006). 17069638 10.1111/j.1365-2583.2006.00682.x PMC1847501

[b68] Nüsslein-Volhard C. & Wieschaus E. Mutations affecting segment number and polarity in *Drosophila*. Nature 287, 795–801 (1980). 6776413 10.1038/287795a0

[b69] Belvin M. P. & Anderson K. V. A conserved signaling pathway: the *Drosophila* toll-dorsal pathway. Annu. Rev. Cell Dev. Biol. 12, 393–416 (1996). 8970732 10.1146/annurev.cellbio.12.1.393

[b70] Kim Y. S. *et al.* Gram-negative bacteria-binding protein, a pattern recognition receptor for lipopolysaccharide and β-1, 3-glucan that mediates the signaling for the induction of innate immune genes in *Drosophila melanogaster* cells. J Biol. Chem. 275, 32721–32727 (2000). 10827089 10.1074/jbc.M003934200

[b71] Matskevich A. A., Quintin J. & Ferrandon D. The *Drosophila* PRR GNBP3 assembles effector complexes involved in antifungal defenses independently of its Toll-pathway activation function. Eur. J. Immunol. 40, 1244–1254 (2010). 20201042 10.1002/eji.200940164 PMC2978882

[b72] Wang Y., Sumathipala N., Rayaprolu S. & Jiang H. Recognition of microbial molecular patterns and stimulation of prophenoloxidase activation by a β-1,3-glucanase-related protein in *Manduca sexta* larval plasma. Insect. Biochem. Molec. 41, 322–331 (2011). 10.1016/j.ibmb.2011.01.010 PMC3066292 21296155

[b73] Ma, C & MR., K. A beta1,3-glucan recognition protein from an insect, *Manduca sexta*, agglutinates microorganisms and activates the phenoloxidase cascade. J. Biol. Chem. 275, 7505–7514 (2000). 10713054 10.1074/jbc.275.11.7505

[b74] Etebari K. *et al.* Deep sequencing-based transcriptome analysis of *Plutella xylostella* larvae parasitized by *Diadegma semiclausum*. BMC Genomics 12, 446 (2011). 21906285 10.1186/1471-2164-12-446 PMC3184118

[b75] Bahrndorff S., Gill C., Lowenberger C., Skovgård H. & Hald B. The effects of temperature and innate immunity on transmission of *Campylobacter jejuni* (Campylobacterales: Campylobacteraceae) between life stages of *Musca domestica* (Diptera: Muscidae). J. Med. Entomol. 51, 670–677 (2014). 24897861 10.1603/me13220

[b76] Tang W. *et al.* DBM-DB: the diamondback moth genome database. Database 2014, bat087; 10.1093/database/bat087 (2014). 24434032 PMC3893660

