# Supplementary material for: Genome-wide characterization and expression profiling of immune genes in the diamondback moth, Plutella xylostella (L.)
Source: Sci Rep. 2015 May 6;5:9877. doi: 10.1038/srep09877 (PMC4421797; doi:10.1038/srep09877)
Supplement: Supplementary Information [file srep09877-s1.doc]

**Supplementary Information**

**Genome-wide characterization and expression profiling of immune genes in the diamondback moth, *Plutella xylostella* (L.)**

Xiaofeng Xia^1,2,3^, Liying Yu^1,2,3^, Minqian Xue^1,2,3^, Xiaoqiang Yu^1,2,4^, Liette Vasseur^1,2,,3,5^, Geoff M. Gurr^1,2,3,6^, Simon W. Baxter^1,2,3,7^, Hailan Lin^1,2,3^, Junhan Lin^1,2,3,8^, and Minsheng You^1,2,3*^

*Correspondence should be addressed to M.Y. (Email: [msyou@iae.fjau.edu.cn](mailto:msyou@iae.fjau.edu.cn)).

^1^Institute of Applied Ecology, Fujian Agriculture and Forestry University, Fuzhou 350002, China.

^2^Fujian-Taiwan Joint Centre for Ecological Control of Crop Pests, Fujian Agriculture and Forestry University, Fuzhou 350002, China

^3^Key Laboratory of Integrated Pest Management for Fujian-Taiwan Crops, Ministry of Agriculture, Fuzhou 350002, China.

^4^School of biological sciences, University of Missouri-Kansas city, Kansas City, Missouri 64110-2499, USA.

^5^Department of Biological Sciences, Brock University, 500 Glenridge Avenue, St. Catharines, Ontario, Canada L2S 3A1.

^6^Graham Centre, Charles Sturt University, Orange, New South Wales 2800, Australia.

^7^School of Molecular & Biomedical Science, the University of Adelaide, Adelaide, South Australia, Australia.

^8^Fujian Vocational College of Bioengineering, Fuzhou 350002, China.

**Table S1 Classification of immune related genes in five insect species**

| Gene family | *D. melanogaster* | *A. gambiae* | *T. castaneum* | *B. mori* | *P. xylostella* |
| --- | --- | --- | --- | --- | --- |
| **Recognition** |  |  |  |  |  |
| PGRP | 13 | 7 | 7 | 12 | 9 |
| GNBP/βGRP | 3 | 7 | 3 | 4 | 18 |
| Galectins | 6 | 8 | 3 | 4 | 4 |
| FREPs | 14 | 61 | 7 | 3 | 2 |
| C-type lectins | 34 | 25 | 16 | 21 | 7 |
| Scavenger receptor A | 5 | 5 | 4 | 4 | 1 |
| Scavenger receptor B | 12 | 15 | 16 | 13 | 13 |
| Scavenger receptor C | 4 | 1 | 1 | 1 | 1 |
| **Signaling** |  |  |  |  |  |
| **Toll Pathway** |  |  |  |  |  |
| Spz | 6 | 6 | 7 | 3 | 5 |
| Toll Receptor | 9 | 10 | 9 | 14 | 9 |
| MyD88 | 1 | 1 | 1 | 1 | 0 |
| Tube | 1 | 1 | 1 | 1 | 1 |
| Pelle | 1 | 1 | 1 | 1 | 3 |
| Cactus | 1 | 1 | 1 | 1 | 1 |
| Dorsal | 2 | 1 | 2 | 1 | 2 |
| TRAF6 | 1 | 1 | 1 | 1 | 2 |
| **IMD Pathway** |  |  |  |  |  |
| IMD | 1 | 1 | 1 | 1 | 1 |
| FADD | 1 | 1 | 1 | 1 | 1 |
| Dredd | 1 | 1 | 1 | 1 | 1 |
| TAK1 | 1 | 1 | 1 | 1 | 1 |
| Tab2 | 1 | 1 | 1 | 1 | 2 |
| IKK-β | 1 | 1 | 2 | 1 | 1 |
| IKK-γ | 1 | 1 | 1 | 1 | 2 |
| Relish | 1 | 1 | 2 | 1 | 3 |
| **JNK Pathway** |  |  |  |  |  |
| JNK | 1 | 1 | 3 | 1 | 1 |
| C-Jun | 1 | 1 | 1 | 1 | 2 |
| Kay | 1 | 1 | 1 | 1 | 1 |
| **JAK-STAT Pathway** |  |  |  |  |  |
| Domeless | 1 | 1 | 1 | 1 | 1 |
| Hopscotch | 1 | 1 | 1 | 0 | 1 |
| STAT | 1 | 2 | 1 | 1 | 2 |
| **Effectors** |  |  |  |  |  |
| PPO | 3 | 9 | 3 | 2 | 1 |
| TEP | 6 | 13 | 4 | 6 | 1 |
| LYS | 13 | 8 | 4 | 4 | 2 |
| AMP | 20 | 10 | 12 | 30 | 7 |
| **Others** |  |  |  |  |  |
| IAP | 4 | 8 | 4 | 5 | 3 |
| Catalase | 2 | 1 | 4 | 7 | 13 |
| Peroxidase | 20 | 26 | 20 | 23 | 17 |
| SOD | 4 | 5 | 4 | 6 | 7 |
| **Total** | **199** | **246** | **153** | **181** | **149** |

***Table S2 Length and location of P. xylostella immune-related genes in different scaffolds***

| **Gene ID** | **Gene name** | **Gene family** | **Length(aa)** | **Scaffold** |
| --- | --- | --- | --- | --- |
| Px015209 | PGRP-SA | PGRP | 176 | scaffold_7:99004:100950:+ |
| Px015207 | PGRP-SA | PGRP | 119 | scaffold_7:90571:92880:+ |
| Px008495 | PGRP-B | PGRP | 210 | scaffold_30:1312930:1314776:- |
| Px008494 | PGRP-D | PGRP | 203 | scaffold_30:1299823:1310882:- |
| Px004942 | PGRP-SA | PGRP | 176 | scaffold_187:374045:380834:+ |
| Px004941 | PGRP-LC | PGRP | 308 | scaffold_187:362917:370434:+ |
| Px004943 | PGRP-2 | PGRP | 216 | scaffold_187:384477:393891:+ |
| Px001311 | PGRP-D | PGRP | 270 | scaffold_116:746839:758656:- |
| Px001312 | PGRP-C | PGRP | 205 | scaffold_116:761353:763802:- |
| Px015066 | βGRP | GNBP/βGRP | 457 | scaffold_689:34030:56084:+ |
| Px015065 | βGRP | GNBP/βGRP | 460 | scaffold_689:29216:32512:+ |
| Px015064 | βGRP | GNBP/βGRP | 437 | scaffold_689:23263:27411:- |
| Px009705 | βGRP | GNBP/βGRP | 475 | scaffold_347:67395:72826:- |
| Px009704 | βGRP | GNBP/βGRP | 452 | scaffold_347:61806:66642:- |
| Px009703 | βGRP | GNBP/βGRP | 491 | scaffold_347:54574:60421:- |
| Px009702 | βGRP | GNBP/βGRP | 426 | scaffold_347:45713:53533:- |
| Px009300 | βGRP | GNBP/βGRP | 390 | scaffold_33:50827:63151:+ |
| Px009301 | βGRP | GNBP/βGRP | 477 | scaffold_33:65252:85557:+ |
| Px009706 | βGRP | GNBP/βGRP | 168 | scaffold_347:73399:74949:+ |
| Px009299 | βGRP | GNBP/βGRP | 512 | scaffold_33:44597:49954:- |
| Px008677 | βGRP | GNBP/βGRP | 437 | scaffold_306:295621:306041:+ |
| Px008676 | βGRP | GNBP/βGRP | 438 | scaffold_306:281086:295332:- |
| Px005587 | βGRP | GNBP/βGRP | 472 | scaffold_200:28753:35149:- |
| Px005586 | βGRP | GNBP/βGRP | 433 | scaffold_200:175:28122:- |
| Px003471 | βGRP | GNBP/βGRP | 474 | scaffold_153:636537:648427:+ |
| Px001059 | βGRP | GNBP/βGRP | 429 | scaffold_1114:15636:18858:- |
| Px001058 | βGRP | GNBP/βGRP | 480 | scaffold_1114:1669:14661:- |
| Px006672 | Galectins | Galectins | 404 | scaffold_233:374297:389578:+ |
| Px011187 | Galectins | Galectins | 338 | scaffold_42:325918:326934:- |
| Px014826 | Galectins | Galectins | 203 | scaffold_660:76308:82508:- |
| Px015997 | Galectins | Galectins | 215 | scaffold_758:23235:41606:- |
| Px001855 | FREPs | FREPs | 445 | scaffold_127:724875:726732:- |
| Px006826 | FREPs | FREPs | 616 | scaffold_24:1313134:1319374:+ |
| Px002615 | C-type lectins | C-type lectins | 220 | scaffold_14:2012018:2018816:- |
| Px002967 | C-type lectins | C-type lectins | 193 | scaffold_147:190998:194139:- |
| Px009511 | C-type lectins | C-type lectins | 167 | scaffold_338:40113:52366:- |
| Px009865 | C-type lectins | C-type lectins | 231 | scaffold_354:85229:90304:+ |
| Px010454 | C-type lectins | C-type lectins | 396 | scaffold_384:328851:357915:+ |
| Px012374 | C-type lectins | C-type lectins | 322 | scaffold_486:66306:69931:- |
| Px012437 | C-type lectins | C-type lectins | 236 | scaffold_49:455443:465954:+ |
| Px016682 | Scavenger receptor A | Scavenger receptor | 300 | scaffold_82:643061:660552:- |
| Px001510 | Scavenger receptor B | Scavenger receptor | 852 | scaffold_12:1879374:1949757:+ |
| Px001589 | Scavenger receptor B | Scavenger receptor | 600 | scaffold_122:58253:73185:- |
| Px004039 | Scavenger receptor B | Scavenger receptor | 512 | scaffold_164:358451:367787:- |
| Px005504 | Scavenger receptor B | Scavenger receptor | 280 | scaffold_20:403:13318:- |
| Px008150 | Scavenger receptor B | Scavenger receptor | 216 | scaffold_290:3790:16539:- |
| Px010290 | Scavenger receptor B | Scavenger receptor | 501 | scaffold_376:106778:154934:+ |
| Px011325 | Scavenger receptor B | Scavenger receptor | 461 | scaffold_428:2158:25650:- |
| Px011335 | Scavenger receptor B | Scavenger receptor | 582 | scaffold_429:13967:26790:- |
| Px011646 | Scavenger receptor B | Scavenger receptor | 319 | scaffold_445:148866:160557:- |
| Px012163 | Scavenger receptor B | Scavenger receptor | 418 | scaffold_475:42022:57503:+ |
| Px012164 | Scavenger receptor B | Scavenger receptor | 495 | scaffold_475:85272:109561:+ |
| Px012922 | Scavenger receptor B | Scavenger receptor | 505 | scaffold_513:2245:16529:- |
| Px015454 | Scavenger receptor B | Scavenger receptor | 512 | scaffold_715:19942:25401:+ |
| Px001649 | Scavenger receptor C | Scavenger receptor | 471 | scaffold_123:136059:144658:+ |
| Px000622 | Spaetzle | Spaetzle | 948 | scaffold_106:381514:396354:- |
| Px004283 | Spaetzle6 | Spaetzle | 207 | scaffold_17:1599620:1602760:- |
| Px015363 | Spaetzle | Spaetzle | 400 | scaffold_703:144966:158365:+ |
| Px003410 | Spaetzle4 | Spaetzle | 513 | scaffold_152:520305:525102:+ |
| Px003237 | Spaetzle3 | Spaetzle | 207 | scaffold_15:2094462:2096809:- |
| Px001789 | Toll6 | Toll receptor | 225 | scaffold_126:234280:234957:+ |
| Px001791 | Toll8 | Toll receptor | 1277 | scaffold_126:488179:492012:+ |
| Px003409 | Toll9 | Toll receptor | 192 | scaffold_152:512127:512705:+ |
| Px004048 | Toll9 | Toll receptor | 733 | scaffold_164:545608:551226:+ |
| Px006338 | Toll | Toll receptor | 413 | scaffold_223:313415:314656:+ |
| Px007264 | Toll10 | Toll receptor | 1345 | scaffold_257:218893:222930:+ |
| Px009819 | Toll | Toll receptor | 803 | scaffold_351:139245:142122:+ |
| Px009907 | Toll9 | Toll receptor | 669 | scaffold_357:934:16075:- |
| Px014888 | Toll10 | Toll receptor | 1336 | scaffold_67:290679:294689:- |
| Px003725 | IRAK4 | IRAK4 (Tube) | 627 | scaffold_16:1488113:1490721:- |
| Px006837 | pelle | pelle | 426 | scaffold_240:268059:276169:- |
| Px015514 | pelle | pelle | 451 | scaffold_72:845461:856594:- |
| Px010499 | pelle | pelle | 411 | scaffold_387:175525:191136:+ |
| Px008096 | TRAF6 | TRAF6 | 402 | scaffold_289:469426:472172:+ |
| Px012423 | TRAF6 | TRAF6 | 501 | scaffold_49:257314:271499:+ |
| Px016665 | Cactus | Cactus | 350 | scaffold_82:213570:223502:+ |
| Px000110 | Dorsal | REL | 734 | scaffold_10:543478:560707:- |
| Px008539 | Dorsal | REL | 339 | scaffold_300:2387:24678:- |
| Px003008 | IMD | IMD | 251 | scaffold_148:80258:82243:- |
| Px011635 | FADD | FADD | 222 | scaffold_444:89170:90015:+ |
| Px000411 | Dredd | Dredd | 884 | scaffold_102:1520912:1533363:- |
| Px002003 | TAK1 | TAK1 | 1135 | scaffold_13:826348:853524:+ |
| Px004524 | TAB2 | TAB | 584 | scaffold_175:1117033:1120906:- |
| Px000611 | TAB2 | TAB | 582 | scaffold_106:76678:80579:+ |
| Px005490 | IKK-β | IKK-β | 769 | scaffold_2:1662671:1664980:- |
| Px004987 | IKK-γ | IKK-γ | 221 | scaffold_188:404439:408300:- |
| Px007462 | IKK-γ | IKK-γ | 651 | scaffold_263:368684:380308:- |
| Px011884 | Relish | REL | 532 | scaffold_458:417:13574:- |
| Px002858 | Relish | REL | 1340 | scaffold_144:134438:179643:- |
| Px009291 | Relish | REL | 1461 | scaffold_329:263235:287027:+ |
| Px016566 | JNK | JNK | 369 | scaffold_802:20525:30478:- |
| Px016832 | c-Jun | c-Jun | 511 | scaffold_84:747723:762514:+ |
| Px004977 | c-Jun | c-Jun | 237 | scaffold_188:237205:237918:+ |
| Px016304 | Kay | Kay | 386 | scaffold_79:744265:747373:+ |
| Px009358 | Domeless | Domeless | 1240 | scaffold_33:1159474:1163196:+ |
| Px013907 | Hopscotch | Hopscotch | 1019 | scaffold_593:71618:99332:- |
| Px017054 | STAT | STAT | 435 | scaffold_878:509:9978:- |
| Px017055 | STAT | STAT | 140 | scaffold_878:10989:19599:- |
| Px013796 | Cecropin | Cecropin | 65 | scaffold_586:104315:104741:+ |
| Px013797 | Cecropin | Cecropin | 65 | scaffold_586:108096:108784:+ |
| Px010113 | Moricin | Moricin | 65 | scaffold_366:124253:124678:+ |
| Px010114 | Moricin | Moricin | 65 | scaffold_366:126990:127838:- |
| Px010115 | Moricin | Moricin | 65 | scaffold_366:131047:131370:- |
| Px000868 | Gloverin | Gloverin | 167 | scaffold_109:2151406:2153612:- |
| Px000869 | Gloverin | Gloverin | 226 | scaffold_109:2163059:2170896:+ |
| Px002706 | Lys2 | Lysozyme | 140 | scaffold_140:665177:666625:+ |
| Px011528 | Lys1 | Lysozyme | 68 | scaffold_44:67601:68182:+ |
| Px002274 | PPO2 | Prophenoloxidase | 709 | scaffold_134:494127:510058:+ |
| Px007031 | PPO-Like | Hexamerin | 763 | scaffold_248:546224:548924:- |
| Px007030 | PPO-Like | Hexamerin | 750 | scaffold_248:540222:543363:+ |
| Px007028 | PPO-Like | Hexamerin | 1207 | scaffold_248:423960:428798:- |
| Px007026 | PPO-Like | Hexamerin | 539 | scaffold_248:410065:412084:- |
| Px007025 | PPO-Like | Hexamerin | 698 | scaffold_248:406804:409828:+ |
| Px007024 | PPO-Like | Hexamerin | 671 | scaffold_248:398782:401656:- |
| Px007023 | PPO-Like | Hexamerin | 711 | scaffold_248:391155:395824:+ |
| Px006820 | PPO-Like | Hexamerin | 736 | scaffold_24:1138827:1141762:- |
| Px000714 | TEP | thioester-containing proteins | 1171 | scaffold_107:612450:626233:+ |
| Px003774 | IAP2 | Inhibitors of Apoptosis | 547 | scaffold_16:2619576:2634061:+ |
| Px004871 | IAP2 | Inhibitors of Apoptosis | 208 | scaffold_185:574097:577363:+ |
| Px004872 | IAP2 | Inhibitors of Apoptosis | 184 | scaffold_185:582849:585715:+ |
| Px017921 | Catalase | Catalase | 515 | scaffold_98:357645:359192:+ |
| Px010516 | Catalase | Catalase | 537 | scaffold_388:93909:95810:+ |
| Px007722 | Catalase | Catalase | 516 | scaffold_274:527381:529099:+ |
| Px007721 | Catalase | Catalase | 481 | scaffold_274:519371:522118:- |
| Px007720 | Catalase | Catalase | 510 | scaffold_274:515989:518509:- |
| Px005629 | Catalase | Catalase | 563 | scaffold_201:365087:366958:+ |
| Px005628 | Catalase | Catalase | 465 | scaffold_201:357248:358645:+ |
| Px005627 | Catalase | Catalase | 495 | scaffold_201:347034:348521:+ |
| Px002300 | Catalase | Catalase | 481 | scaffold_135:20256:21701:- |
| Px001209 | Catalase | Catalase | 535 | scaffold_114:851913:853847:+ |
| Px000982 | Catalase | Catalase | 510 | scaffold_110:349846:352304:+ |
| Px000981 | Catalase | Catalase | 483 | scaffold_110:346929:348380:+ |
| Px000980 | Catalase | Catalase | 516 | scaffold_110:342495:344211:- |
| Px016786 | Peroxidases | Peroxidases | 564 | scaffold_831:33446:47151:+ |
| Px015891 | Peroxidases | Peroxidases | 759 | scaffold_75:211996:224483:- |
| Px015890 | Peroxidases | Peroxidases | 708 | scaffold_75:124769:149268:- |
| Px004768 | Peroxidases | Peroxidases | 585 | scaffold_181:444797:472171:- |
| Px013761 | Peroxidases | Peroxidases | 680 | scaffold_581:88897:95664:+ |
| Px012442 | Peroxidases | Peroxidases | 608 | scaffold_49:521210:527509:+ |
| Px010494 | Peroxidases | Peroxidases | 226 | scaffold_387:109331:112418:+ |
| Px009205 | Peroxidases | Peroxidases | 399 | scaffold_325:169784:191651:- |
| Px008866 | Peroxidases | Peroxidases | 849 | scaffold_310:162590:192532:+ |
| Px007408 | Peroxidases | Peroxidases | 190 | scaffold_261:144706:151308:+ |
| Px007394 | Peroxidases | Peroxidases | 1039 | scaffold_260:351819:374887:+ |
| Px007137 | Peroxidases | Peroxidases | 1072 | scaffold_250:154052:170775:+ |
| Px008919 | Peroxidases | Peroxidases | 288 | scaffold_312:85237:89579:- |
| Px015745 | Peroxidases | Peroxidases | 782 | scaffold_737:63243:70453:- |
| Px016843 | Peroxidases | Peroxidases | 202 | scaffold_84:904613:905622:+ |
| Px005951 | Peroxidases | Peroxidases | 169 | scaffold_210:426097:431432:+ |
| Px004840 | Peroxidases | Peroxidases | 627 | scaffold_184:357709:364058:- |
| Px018001 | Cu/Zn SOD | Superoxide dismutase | 151 | scaffold_99:422442:425315:+ |
| Px017667 | Mn-Fe SOD | Superoxide dismutase | 175 | scaffold_95:207026:210050:- |
| Px015572 | Cu/Zn SOD | Superoxide dismutase | 138 | scaffold_725:58644:64152:+ |
| Px017040 | Cu/Zn SOD | Superoxide dismutase | 353 | scaffold_872:23651:30626:+ |
| Px010649 | Cu/Zn SOD | Superoxide dismutase | 802 | scaffold_391:323885:346033:- |
| Px001607 | Cu/Zn SOD | Superoxide dismutase | 353 | scaffold_122:349618:358266:+ |
| Px007001 | Mn-Fe SOD | Superoxide dismutase | 175 | scaffold_248:103425:107962:- |

**Table S3 Matrix of protein sequence identity of *P. xylostella* βGRPs**

| Seq-> | GRP1 | GRP2 | GRP3 3 | GRP4 4 | GRP5 5 | GRP6 6 | GRP7 7 | GRP8 8 | GRP9 9 | GRP10 1110 | GRP11 11 | GRP12 | GRP13 | GRP14 | GRP15 | GRP16 | GRP17 | GRP18 |
| --- | --- | --- | --- | --- | --- | --- | --- | --- | --- | --- | --- | --- | --- | --- | --- | --- | --- | --- |
| Px001058(GRP1) | ID | 0.471 | 0.356 | 0.367 | 0.363 | 0.360 | 0.364 | 0.231 | 0.272 | 0.498 | 0.860 | 0.528 | 0.396 | 0.362 | 0.076 | 0.364 | 0.360 | 0.384 |
| Px001059(GRP2） | 0.471 | ID | 0.346 | 0.419 | 0.369 | 0.424 | 0.486 | 0.249 | 0.302 | 0.850 | 0.412 | 0.861 | 0.394 | 0.439 | 0.073 | 0.484 | 0.385 | 0.398 |
| Px003471(GRP3) | 0.356 | 0.346 | ID | 0.761 | 0.380 | 0.374 | 0.377 | 0.224 | 0.300 | 0.342 | 0.300 | 0.365 | 0.398 | 0.421 | 0.056 | 0.377 | 0.380 | 0.804 |
| Px005586(GRP4) | 0.367 | 0.419 | 0.761 | ID | 0.399 | 0.442 | 0.465 | 0.234 | 0.302 | 0.379 | 0.310 | 0.380 | 0.384 | 0.443 | 0.060 | 0.460 | 0.412 | 0.903 |
| Px005587(GRP5) | 0.363 | 0.369 | 0.380 | 0.399 | ID | 0.765 | 0.416 | 0.232 | 0.354 | 0.379 | 0.300 | 0.390 | 0.431 | 0.456 | 0.058 | 0.420 | 0.917 | 0.411 |
| Px008676(GRP6) | 0.360 | 0.424 | 0.374 | 0.442 | 0.765 | ID | 0.461 | 0.241 | 0.326 | 0.390 | 0.296 | 0.392 | 0.413 | 0.440 | 0.065 | 0.461 | 0.781 | 0.435 |
| Px008677(GRP7) | 0.364 | 0.486 | 0.377 | 0.465 | 0.416 | 0.461 | ID | 0.246 | 0.346 | 0.445 | 0.323 | 0.444 | 0.436 | 0.492 | 0.072 | 0.944 | 0.432 | 0.451 |
| Px009299(GRP8) | 0.231 | 0.249 | 0.224 | 0.234 | 0.232 | 0.241 | 0.246 | ID | 0.144 | 0.231 | 0.198 | 0.243 | 0.217 | 0.221 | 0.352 | 0.250 | 0.241 | 0.236 |
| Px009300(GRP9) | 0.272 | 0.302 | 0.300 | 0.302 | 0.354 | 0.326 | 0.346 | 0.144 | ID | 0.319 | 0.237 | 0.341 | 0.495 | 0.631 | 0.074 | 0.346 | 0.350 | 0.316 |
| Px009301(GRP10) | 0.498 | 0.850 | 0.342 | 0.379 | 0.379 | 0.390 | 0.445 | 0.231 | 0.319 | ID | 0.450 | 0.869 | 0.407 | 0.439 | 0.079 | 0.445 | 0.377 | 0.370 |
| Px009702(GRP11) | 0.860 | 0.412 | 0.300 | 0.310 | 0.300 | 0.296 | 0.323 | 0.198 | 0.237 | 0.450 | ID | 0.468 | 0.354 | 0.311 | 0.084 | 0.321 | 0.298 | 0.330 |
| Px009703(GRP12) | 0.528 | 0.861 | 0.365 | 0.380 | 0.390 | 0.392 | 0.444 | 0.243 | 0.341 | 0.869 | 0.468 | ID | 0.429 | 0.464 | 0.077 | 0.444 | 0.388 | 0.386 |
| Px009704(GRP13) | 0.396 | 0.394 | 0.398 | 0.384 | 0.431 | 0.413 | 0.436 | 0.217 | 0.495 | 0.407 | 0.354 | 0.429 | ID | 0.464 | 0.068 | 0.432 | 0.432 | 0.408 |
| Px009705(GRP14) | 0.362 | 0.439 | 0.421 | 0.443 | 0.456 | 0.440 | 0.492 | 0.221 | 0.631 | 0.439 | 0.311 | 0.464 | 0.464 | ID | 0.061 | 0.492 | 0.457 | 0.456 |
| Px009706(GRP15) | 0.076 | 0.073 | 0.056 | 0.060 | 0.058 | 0.065 | 0.072 | 0.352 | 0.074 | 0.079 | 0.084 | 0.077 | 0.068 | 0.061 | ID | 0.072 | 0.061 | 0.058 |
| Px015064(GRP16) | 0.364 | 0.484 | 0.377 | 0.460 | 0.420 | 0.461 | 0.944 | 0.250 | 0.346 | 0.445 | 0.321 | 0.444 | 0.432 | 0.492 | 0.072 | ID | 0.438 | 0.449 |
| Px015065(GRP17) | 0.360 | 0.385 | 0.380 | 0.412 | 0.917 | 0.781 | 0.432 | 0.241 | 0.350 | 0.377 | 0.298 | 0.388 | 0.432 | 0.457 | 0.061 | 0.438 | ID | 0.427 |
| Px015066(GRP18) | 0.384 | 0.398 | 0.804 | 0.903 | 0.411 | 0.435 | 0.451 | 0.236 | 0.316 | 0.370 | 0.330 | 0.386 | 0.408 | 0.456 | 0.058 | 0.449 | 0.427 | ID |

**Table S4 Primers used for q**RT-PCR

| Gene ID | Gene name | Forward | Reverse |
| --- | --- | --- | --- |
| Px013797  Px010115  Px000868  Px002706  Px002274  Control | Cecropin  Moricin  Gloverin  Lysozyme  PPO  RISC | 5'-TTCGTGTTCGTGGCTGTTTT-3'  5'-TTTTGATGCTGGCTCTGGTG-3'  5'-AGGTGAATTGACGGAACCAGA-3'  5'-AGTTGATAACTGACGACATCACGAA -3'  5'-GTGGAGGAGAAATGTGGAGGAG-3'  5'-AATCAGGCCAATTTACCGC-3' | 5'- GGCCAACTCTTTCCAATTTCTT-3'  5'-TTGAGGGCGTTGACGTTG-3'  5'-AGATGTCCTTGAGTGGCGAAA-3'  5'-GGCACTTGTTCTTCCATCCATAC-3'  5'-GGAGTGTCCGTTGTTGTGGA-3'  5'-CTGGGTTTACGCCAGTTACG -3' |


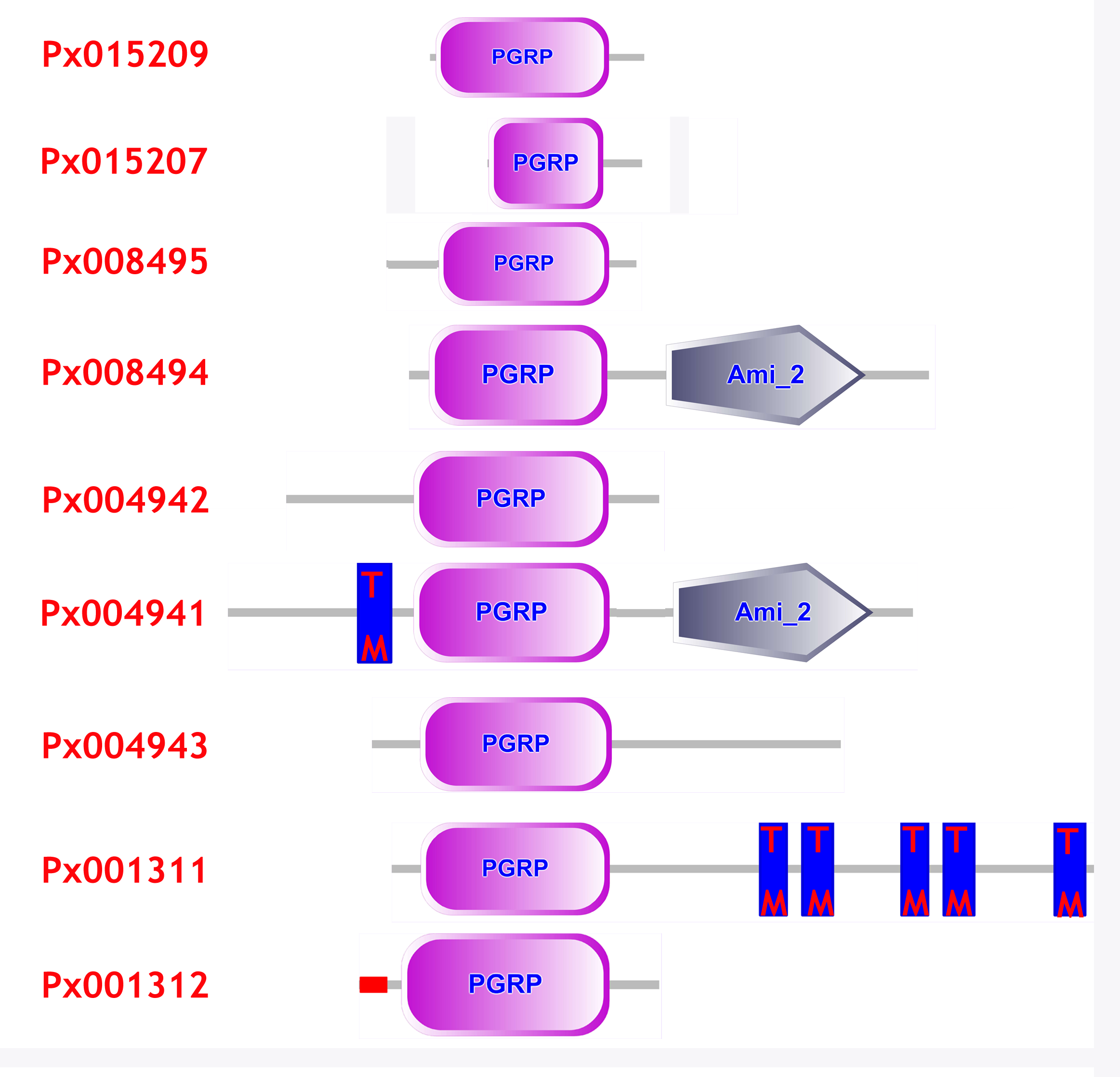


**Fig. S1 The domain architecture of *P. xylostella* PGRPs.** Signal peptides were indicated by red lines (Px001312), the transmembrane domains were indicated by dark blue boxes, the PGRP domains were indicated by pink boxes, and the possible amidase domains were also indicated in the figures by pentagon boxes.


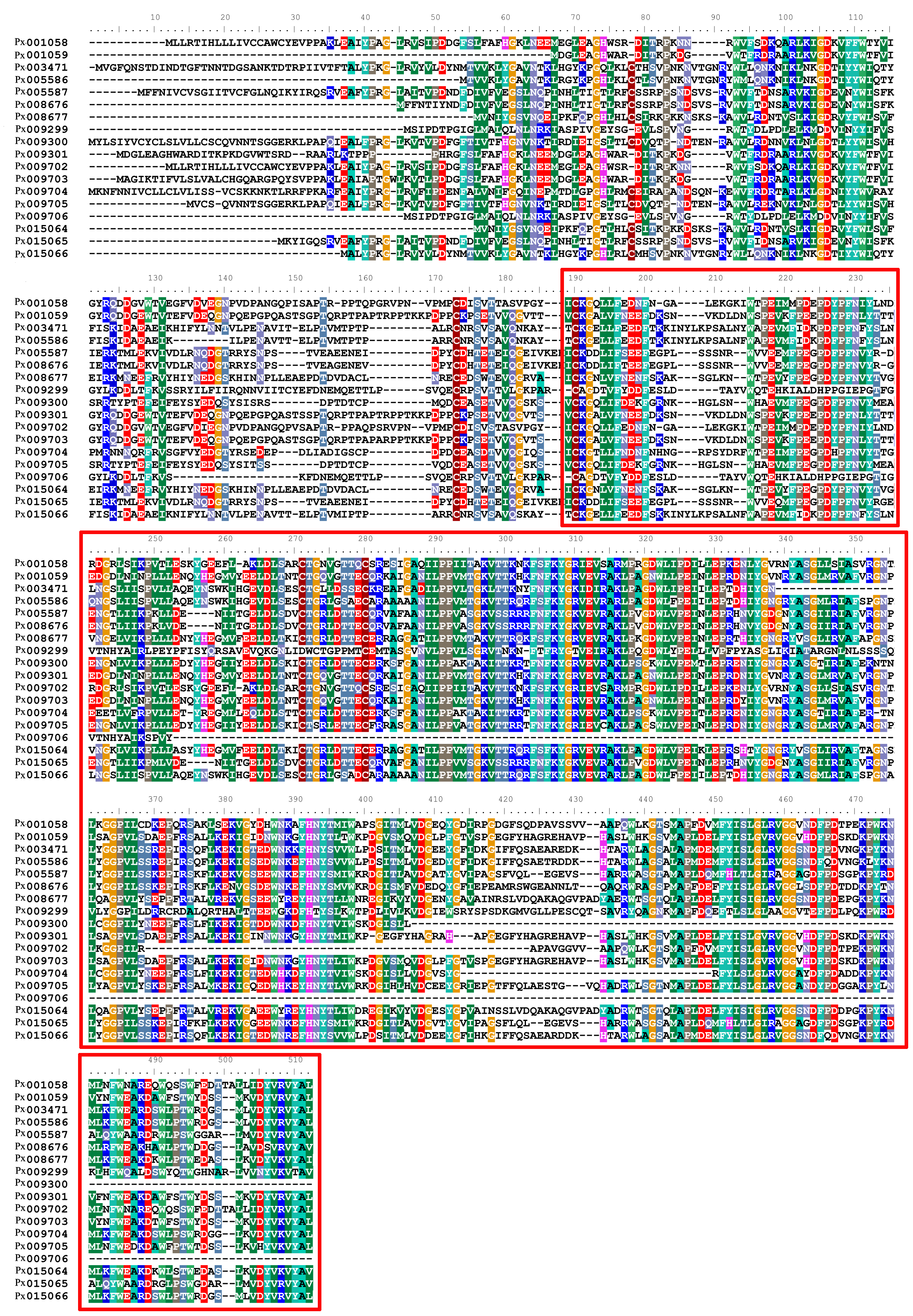


**Fig. S2 Multiple protein alignment of *P. xylostella* βGRPs.** The red box represents the βGRP conserved domain of glycosyl hydrolase family 16.


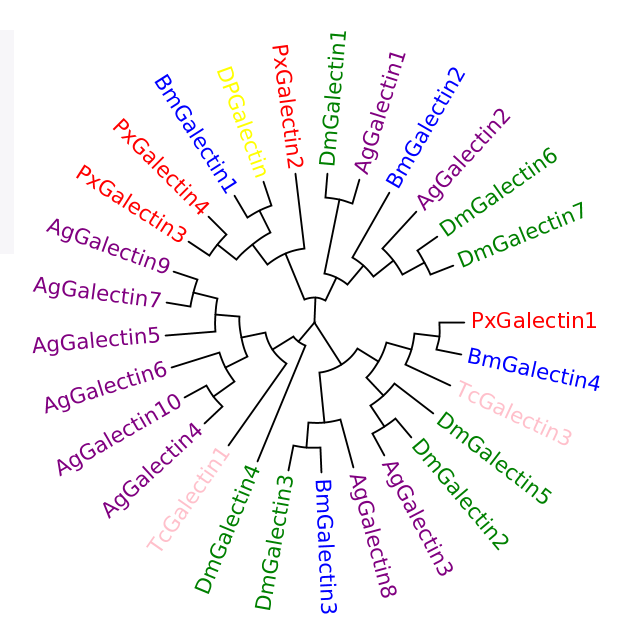


**Fig. S3 Phylogenetic analysis of galectins based on the sequences from *Danaus plexippus* (Dp), *Bombyx mori* (Bm)*, Plutella xylostella* (Px), *Drosophila melanogaster* (Dm), *Anopheles gambiae* (Ag), and *Tribolium castaneum* (Tc).**


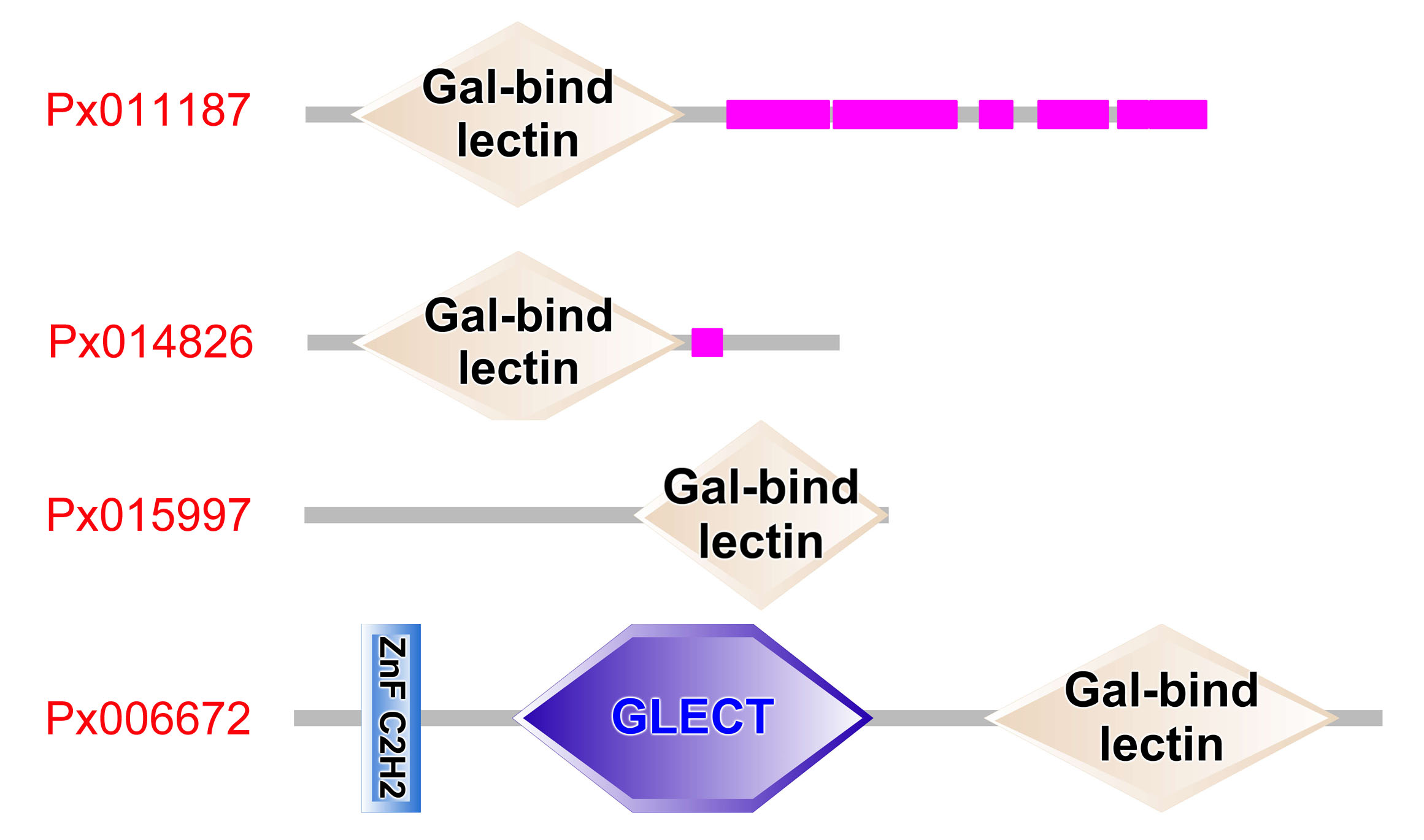


**Fig. S4 Domain architecture of the *P. xylostella* galectins.** The pink boxes denote **g**alectin domains (Gal-bind lectins) with carbohydrate recognition domain (CRD), the purple hexagon represents GLECT domain with CRD of the galectin, and the red rectangle indicates the low complexity region.

.


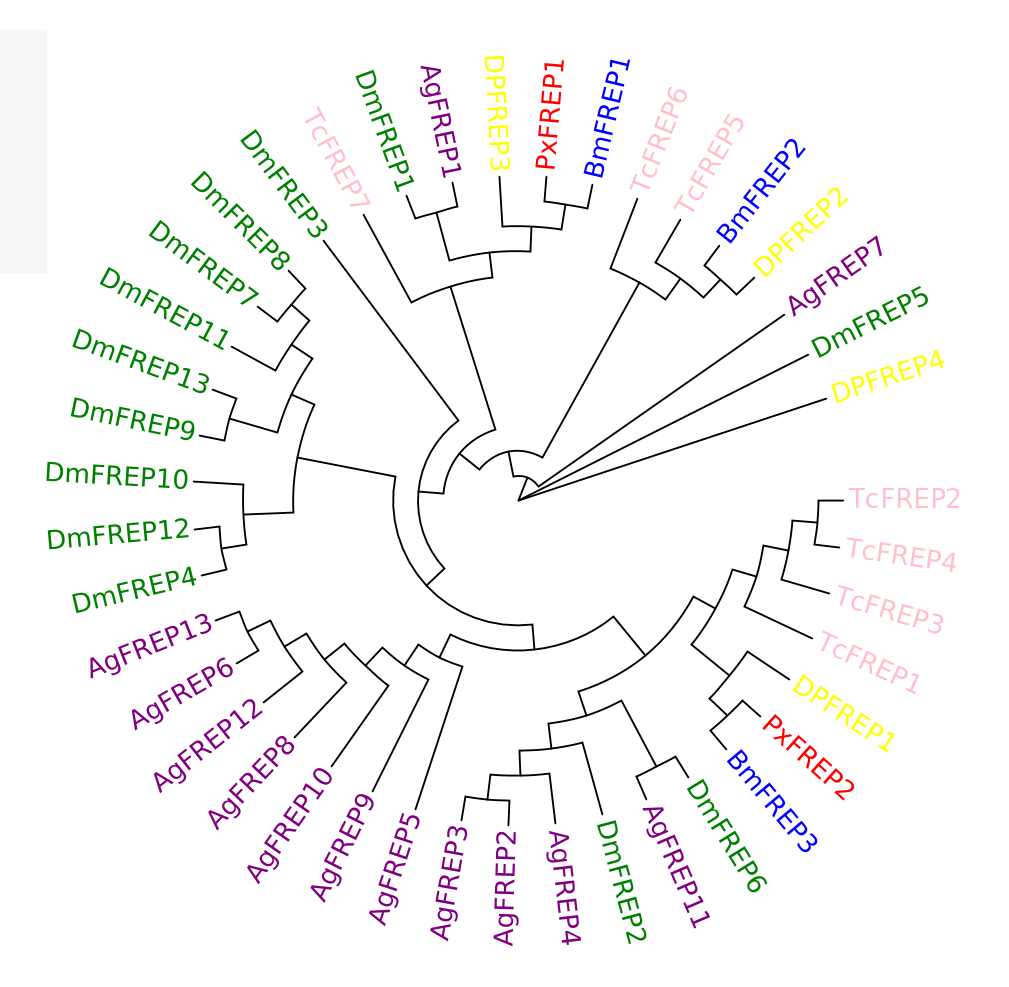


**Fig. S5 Phylogenetic analysis of fibrinogen-related proteins (FREPs) based on the sequences from *Danaus plexippus* (Dp), *Bombyx mori* (Bm)*, Plutella xylostella* (Px), *Drosophila melanogaster* (Dm), *Anopheles gambiae* (Ag), and *Tribolium castaneum* (Tc).**

**
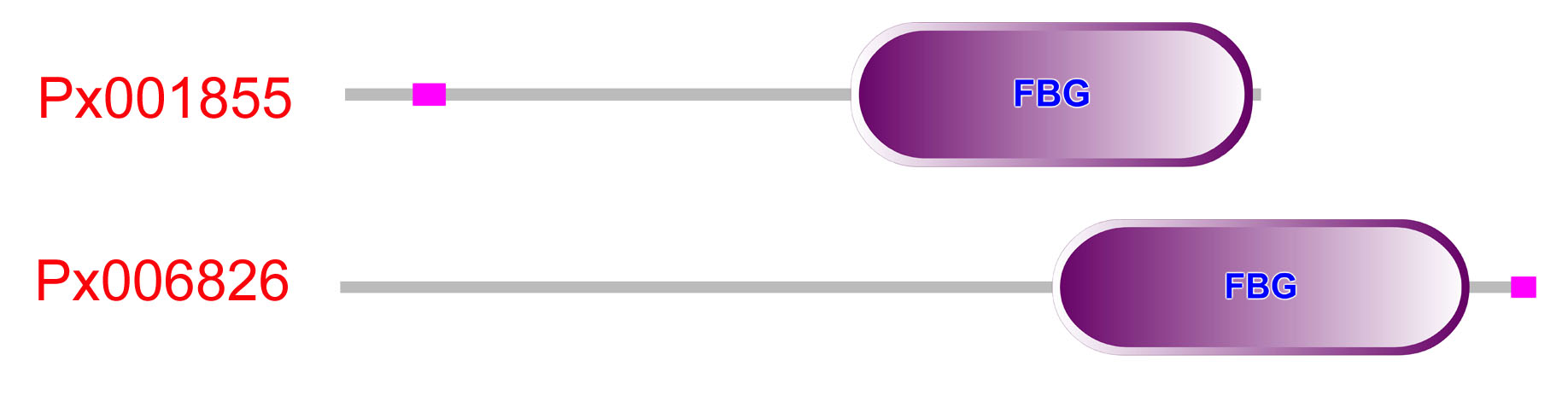
**

**Fig. S6 Domain architecture of the *P. xylostella* fibrinogen-related proteins.** The purple boxes denote the FREP domains, and the red rectangle represents the low complexity region.


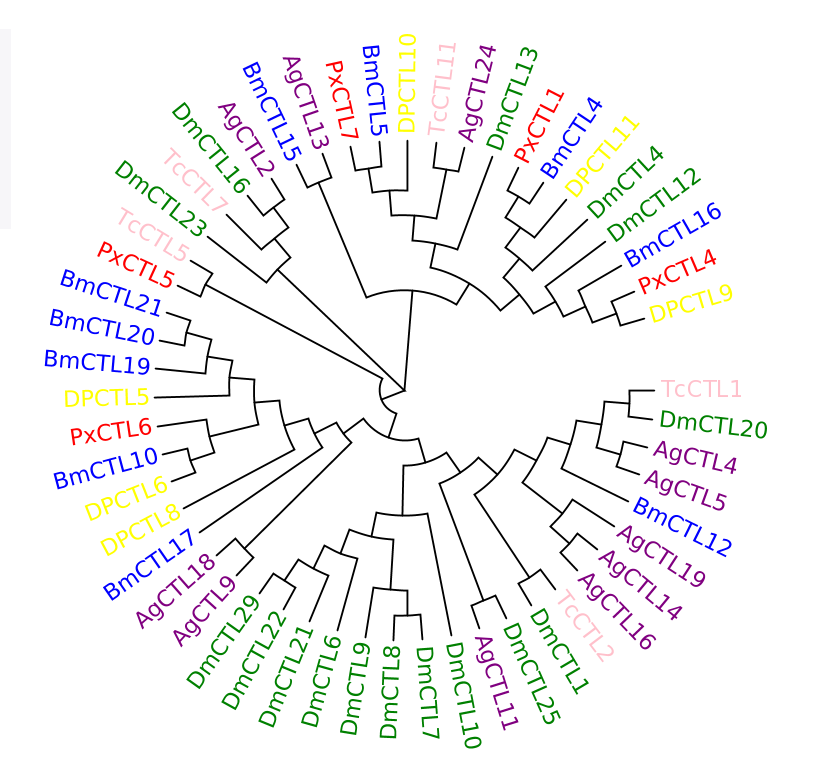


**Fig. S7 Phylogenetic analysis of C-type lectins based on the sequences from *Danaus plexippus* (Dp), *Bombyx mori* (Bm)*, Plutella xylostella* (Px), *Drosophila melanogaster* (Dm), *Anopheles gambiae* (Ag), and *Tribolium castaneum* (Tc).**


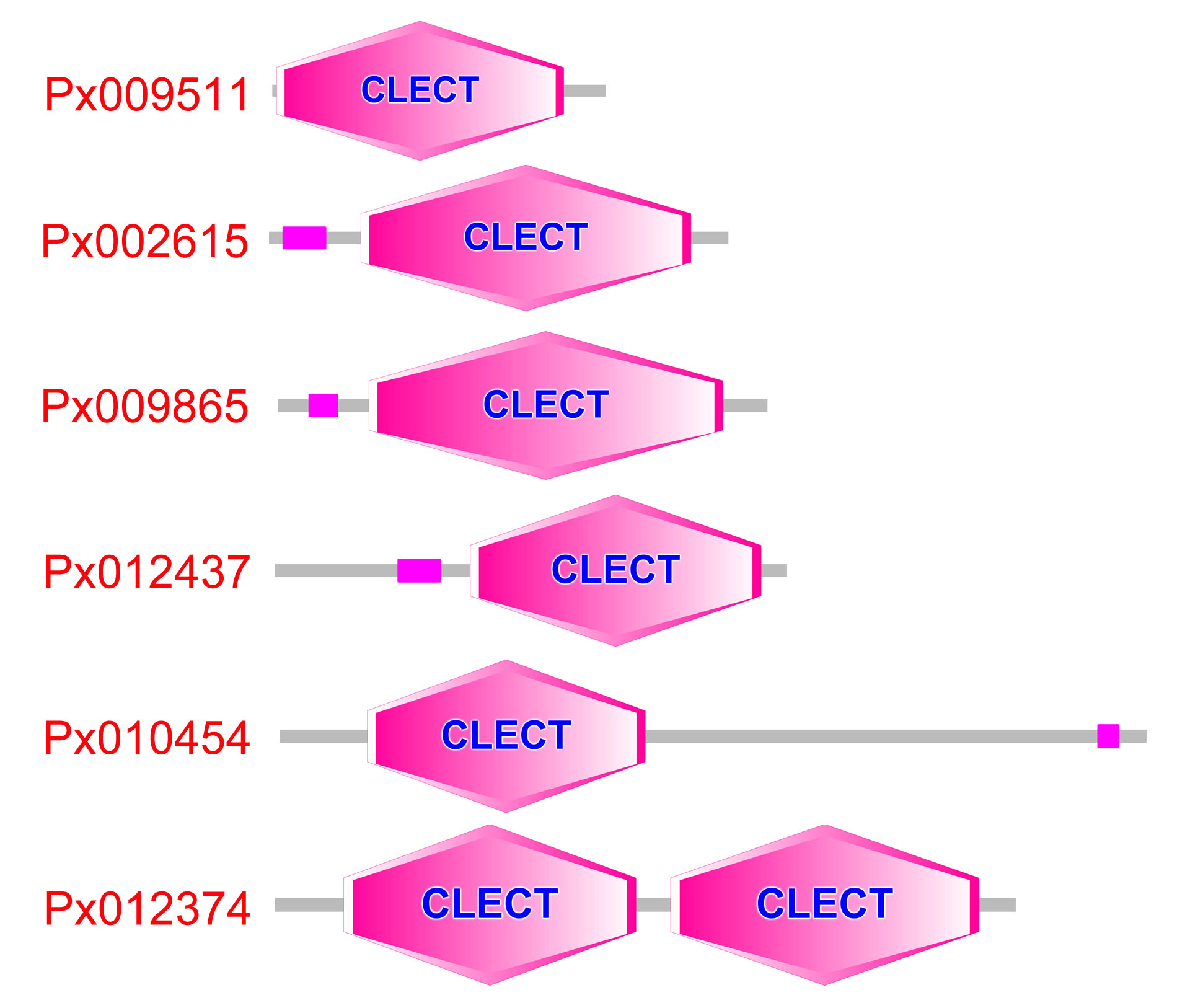


**Fig. S8 Domain architecture of the *P. xylostella* C-type lectins.** The red hexagonal box indicates the C-type lectins domains, and the red rectangle denotes the low complexity region.


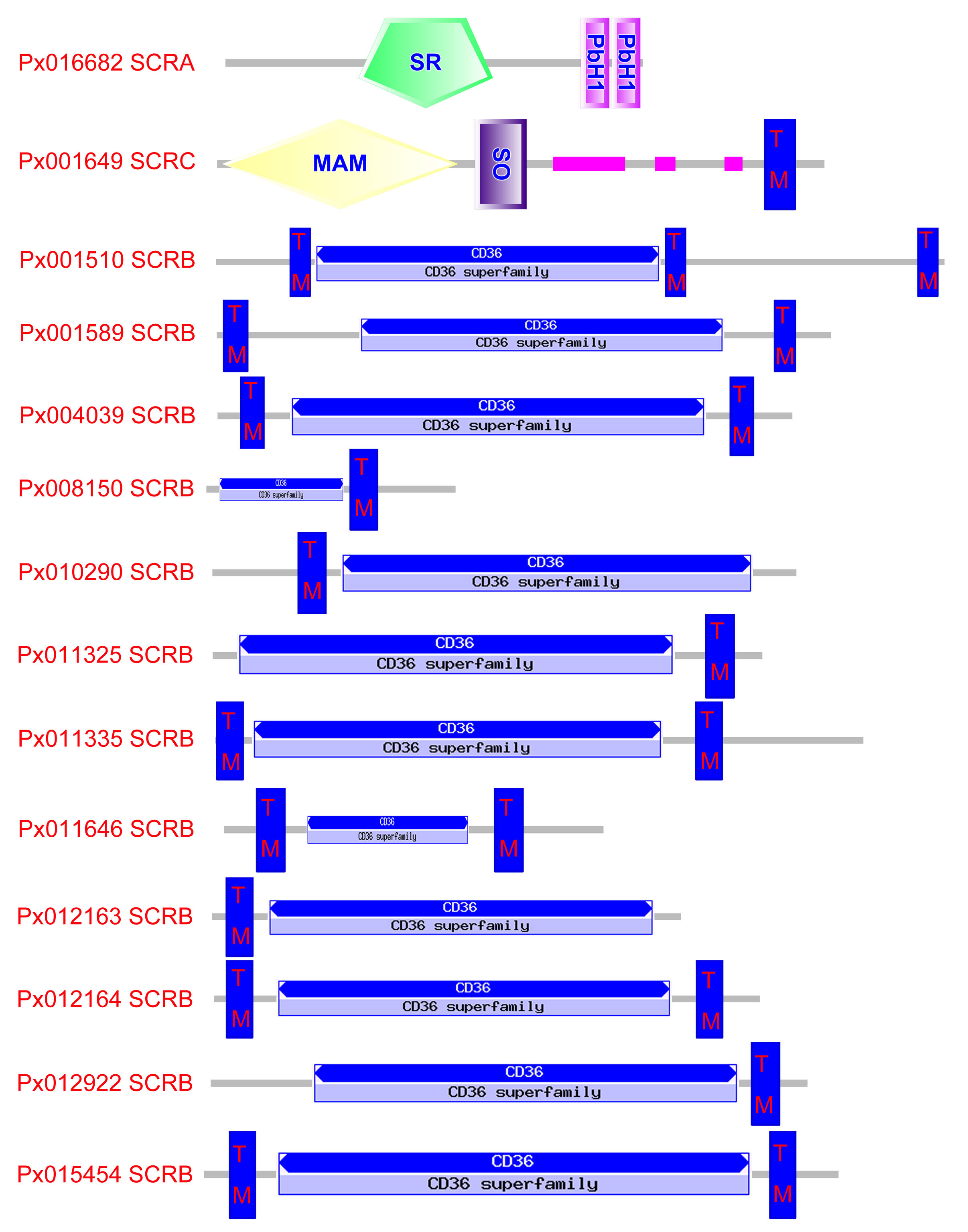


**Fig. S9 Domain architecture of the *P. xylostella* scavenger receptors.** SR denotes the scavenger receptor Cysteine-Rich (SRCR) domain in the subfamily of scavenger receptor A, MAM denotes the conserved domains of Meprin A5 antigen and RPTP Mu, SO denotes somatomedin-B-like in SRCC, CD36 is the conserved domain in SRCB, and TM is the transmembrane region in the sequences.


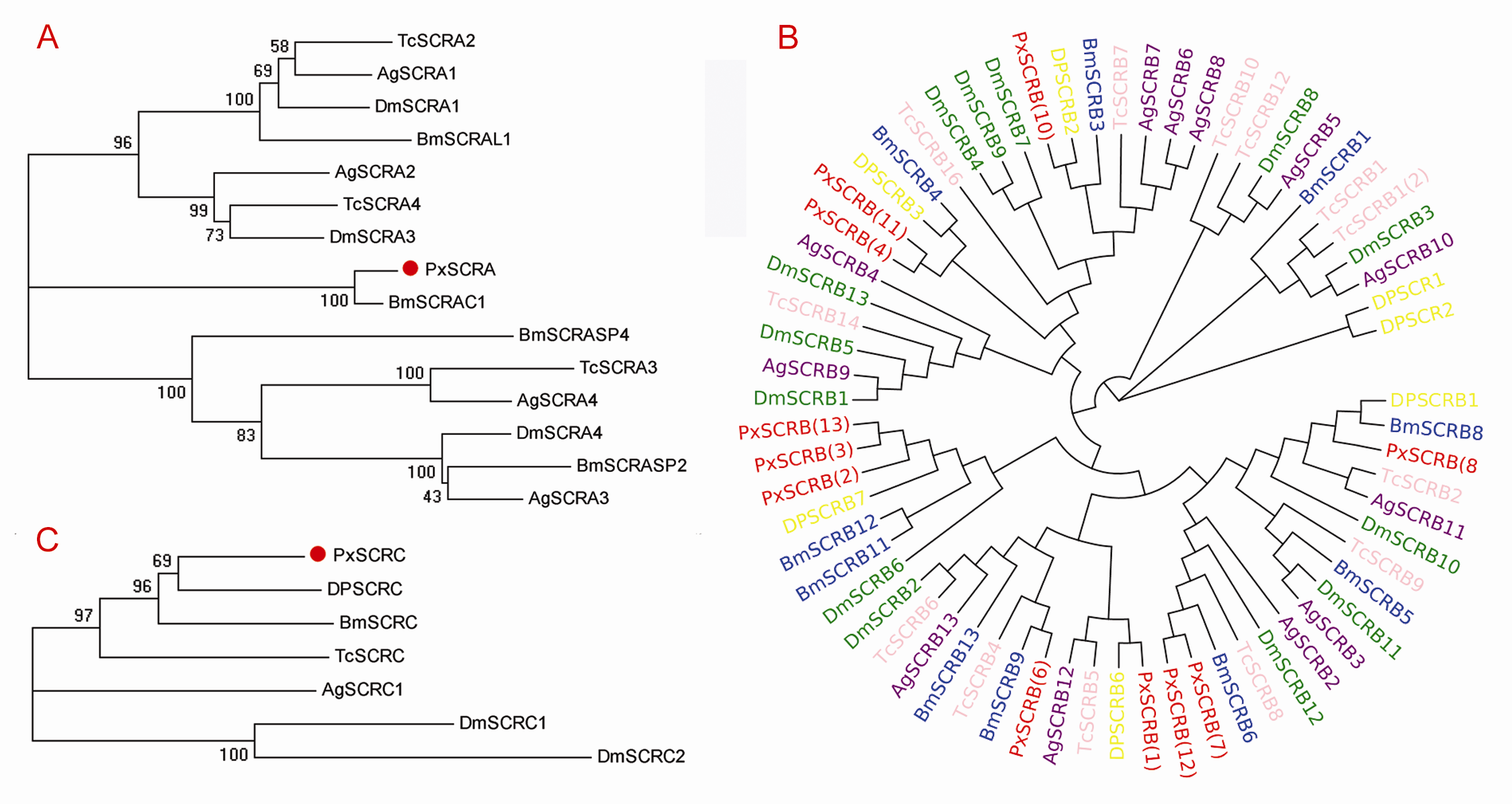


**Fig. S10 Phylogenetic analysis of scavenger receptors based on the sequences from *Danaus plexippus* (Dp), *Bombyx mori* (Bm)*, Plutella xylostella* (Px), *Drosophila melanogaster* (Dm), *Anopheles gambiae* (Ag), and *Tribolium castaneum* (Tc).**


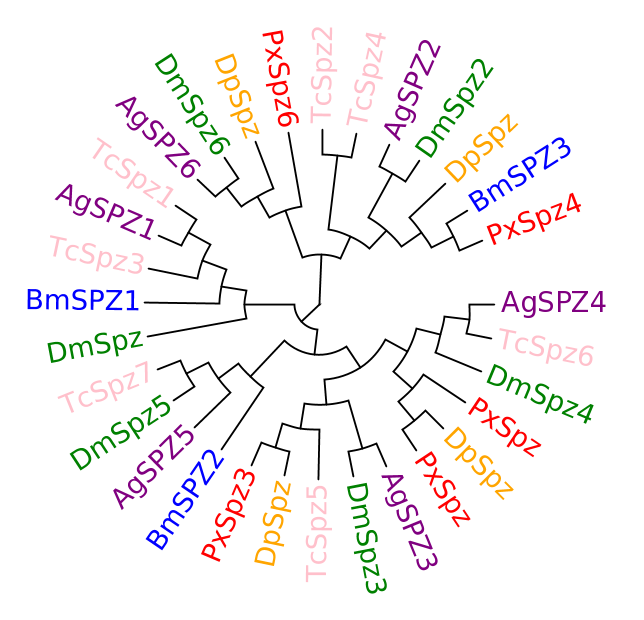


**Fig. S11 Phylogenetic analysis of SPZs based on the sequences from *Danaus plexippus* (Dp), *Bombyx mori* (Bm)*, Plutella xylostella* (Px), *Drosophila melanogaster* (Dm), *Anopheles gambiae* (Ag), and *Tribolium castaneum* (Tc).**


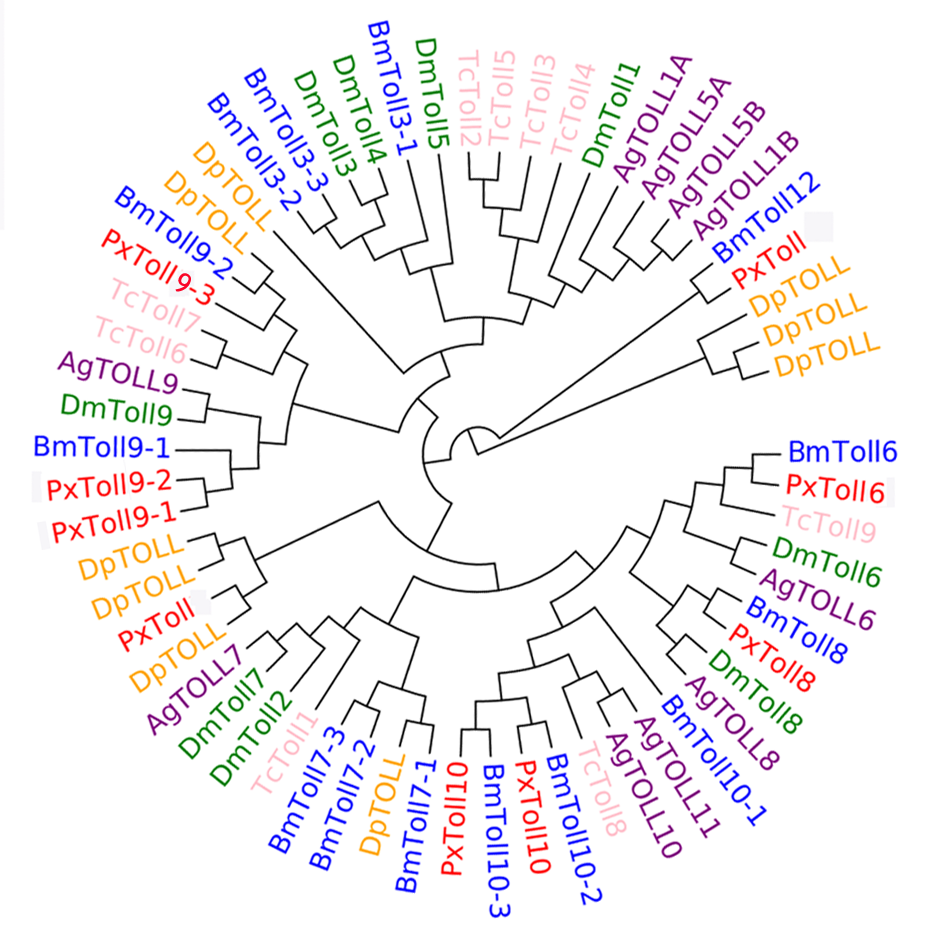


**Fig. S12 Phylogenetic analysis of Toll receptors based on the sequences from *D. plexippus* (Dp), *B. mori* (Bm)*, P. xylostella* (Px), *D. melanogaster* (Dm), *A. gambiae* (Ag), and *T. castaneum* (Tc).**


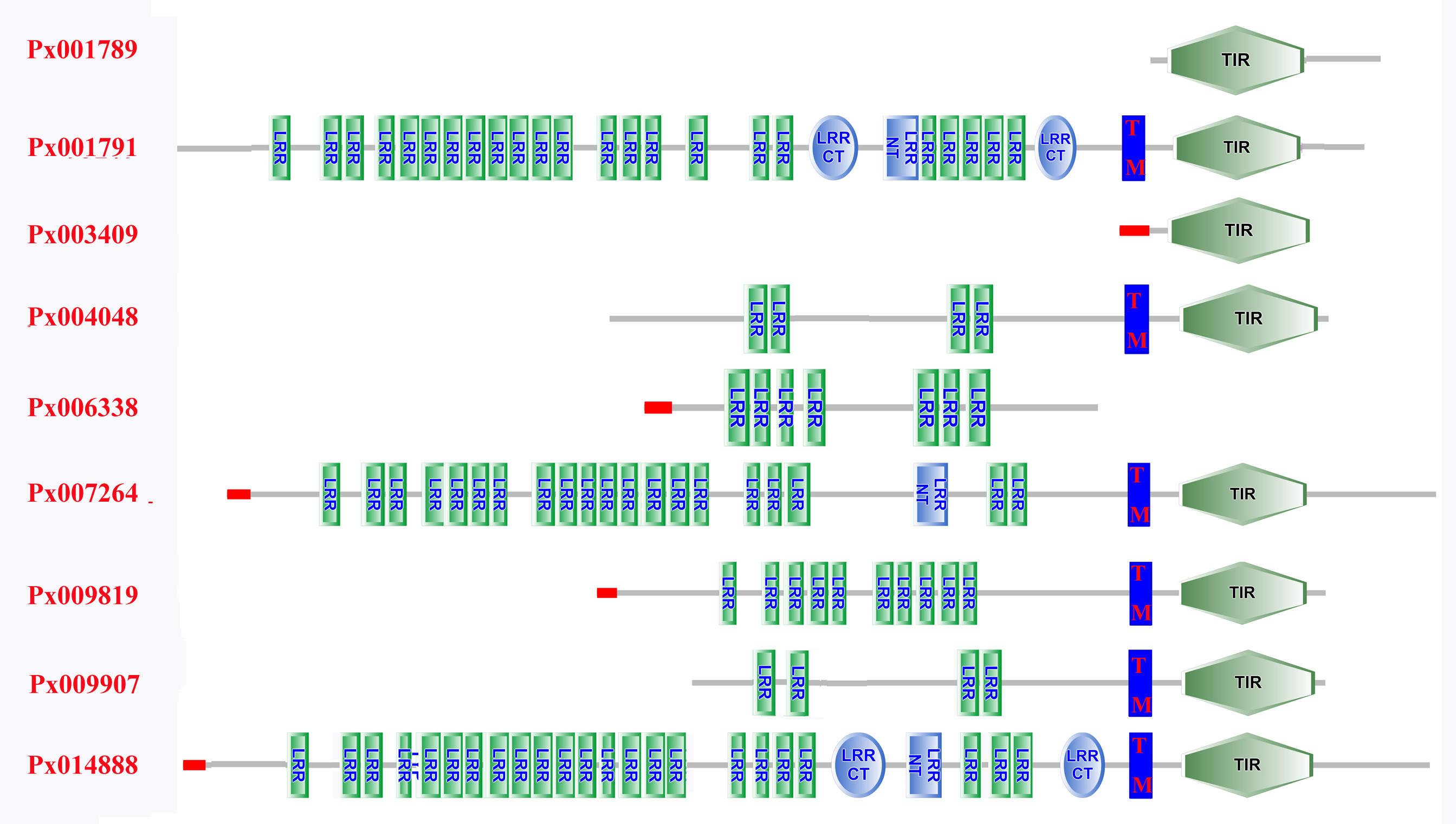


**Fig. S13 The domain architecture of *P. xylostella* Toll receptors.** Signal peptides were indicated by red lines, LRR indicated leucine rich repeat, LRR-CT for C-terminal LRR domain, LRR-NT for N-terminal LRR domain, TM indicated transmembrane domain, and TIR is for the Toll/interleukin-1 receptor (IL-1R) homologous region.


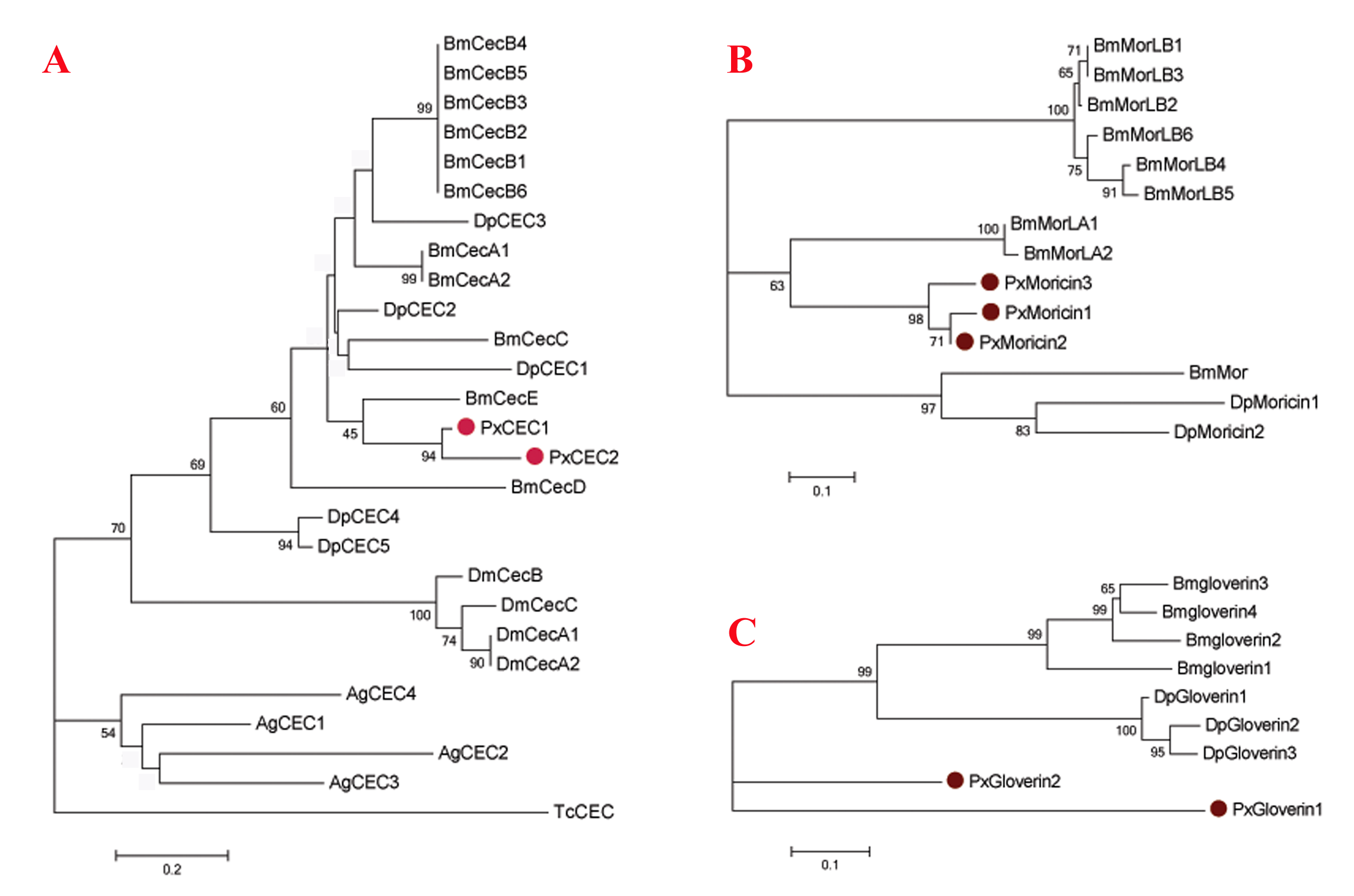


**Fig. S14 Phylogenetic analysis of AMPs based on the sequences from *D. plexippus* (Dp), *B. mori* (Bm)*, P. xylostella* (Px), *T. castaneum* (Tc), *D. melanogaster* (Dm) and *A. gambiae* (Ag).** Phlylogenetic analysis of Cecropin (A), Moricin (B), and Gloverin (C).


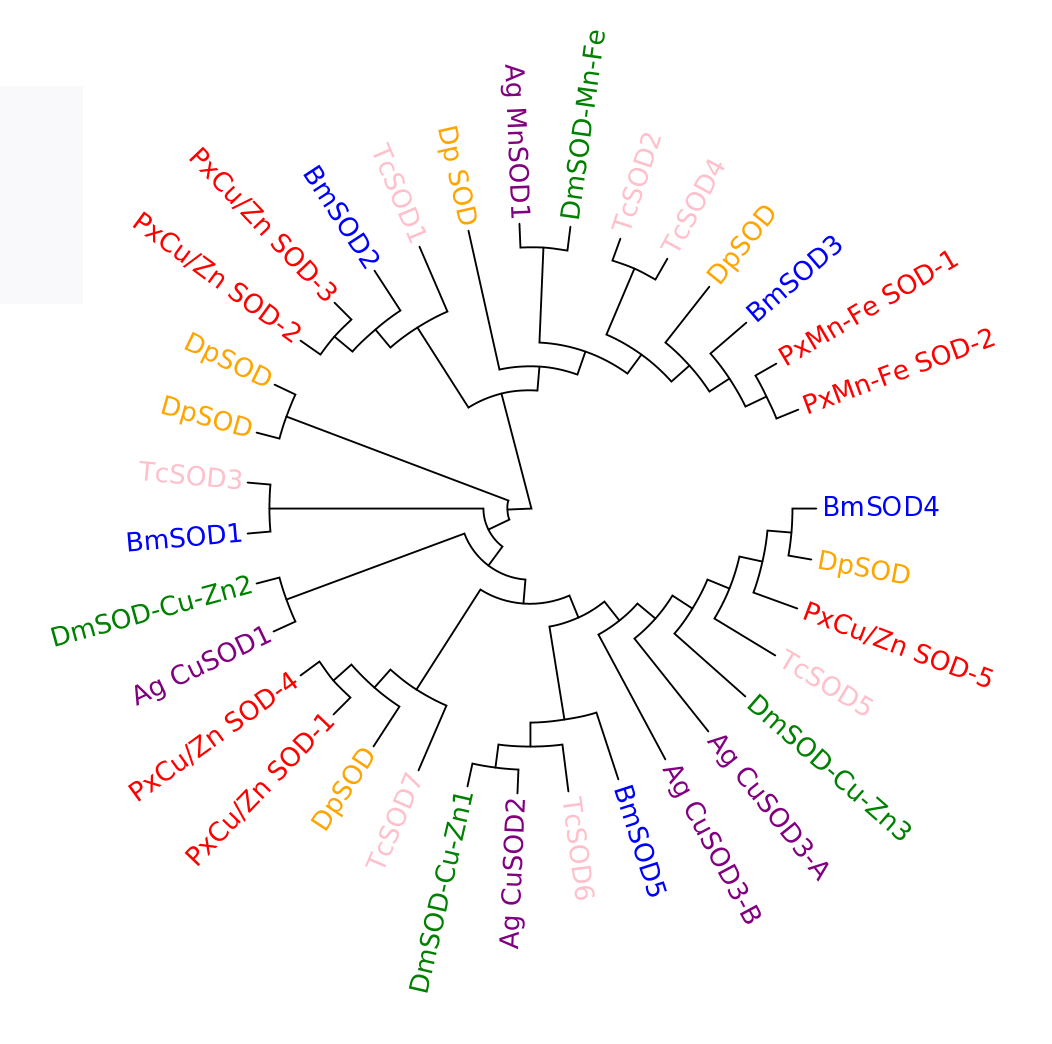


**Fig. S15 Phylogenetic analysis of SODs based on the sequences from *D. plexippus* (Dp), *B. mori* (Bm)*, P. xylostella* (Px), *D. melanogaster* (Dm), *A. gambiae*(Ag), and *T. castaneum* (Tc).**


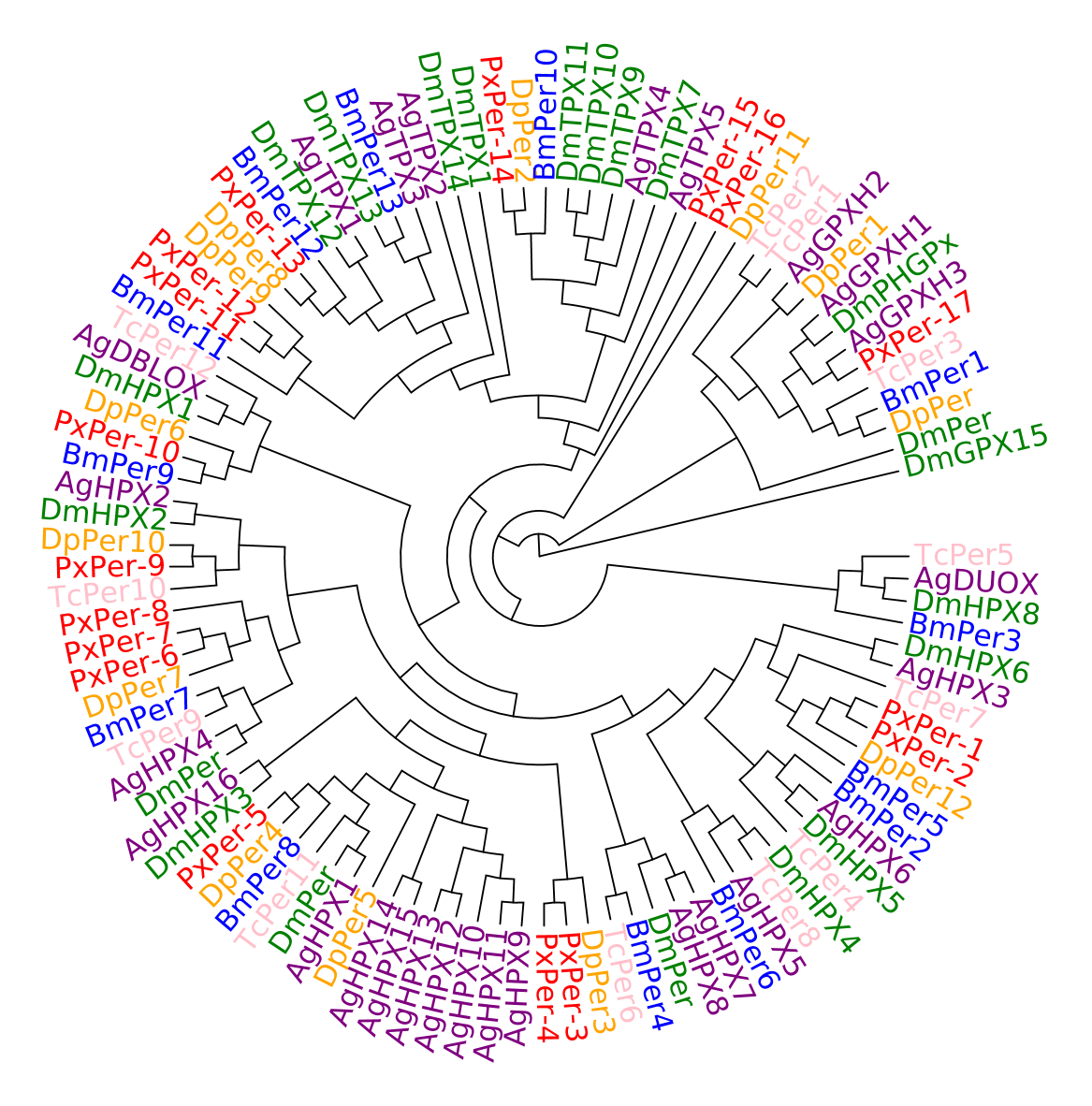


**Fig. S16 Phylogenetic analysis of peroxidase based on the sequences from *D. plexippus* (Dp), *B. mori* (Bm)*, P. xylostella* (Px), *D. melanogaster* (Dm), *A. gambiae* (Ag), and *T. castaneum* (Tc).**


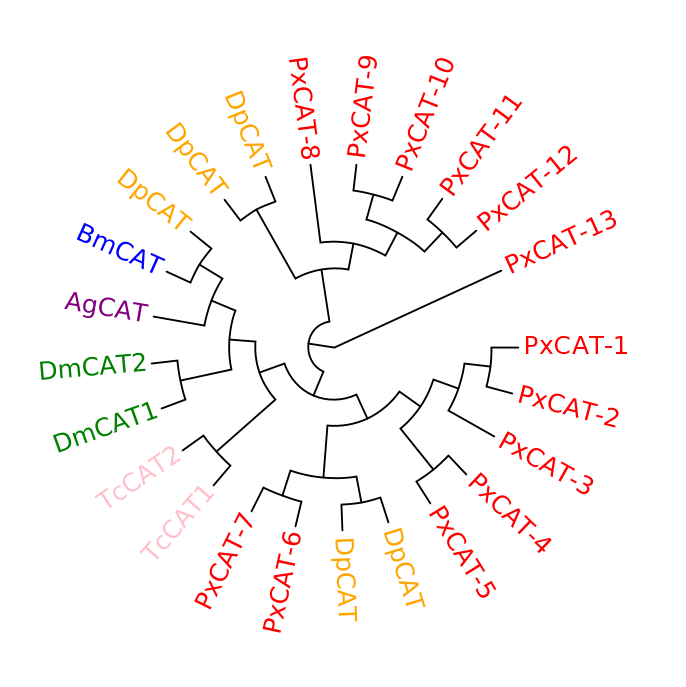


**Fig. S17 Phylogenetic analysis of catalases (CATs) based on the sequences from *D. plexippus* (Dp), *B. mori* (Bm)*, P. xylostella* (Px), *D. melanogaster* (Dm), *A. gambiae* (Ag), and *T. castaneum* (Tc).**


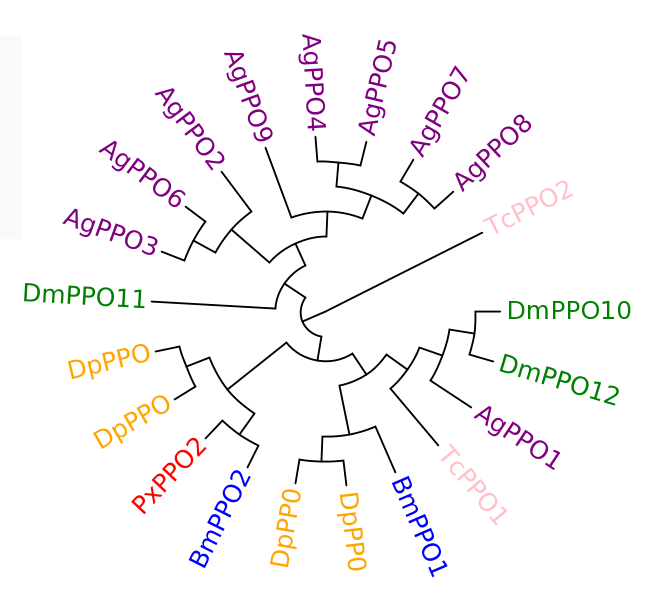


**Fig. S18 Phylogenetic analysis of PPO based on the sequences from *D. plexippus* (Dp), *B. mori* (Bm)*, P. xylostella* (Px), *D. melanogaster* (Dm), *A. gambiae* (Ag), and *T. castaneum* (Tc).**


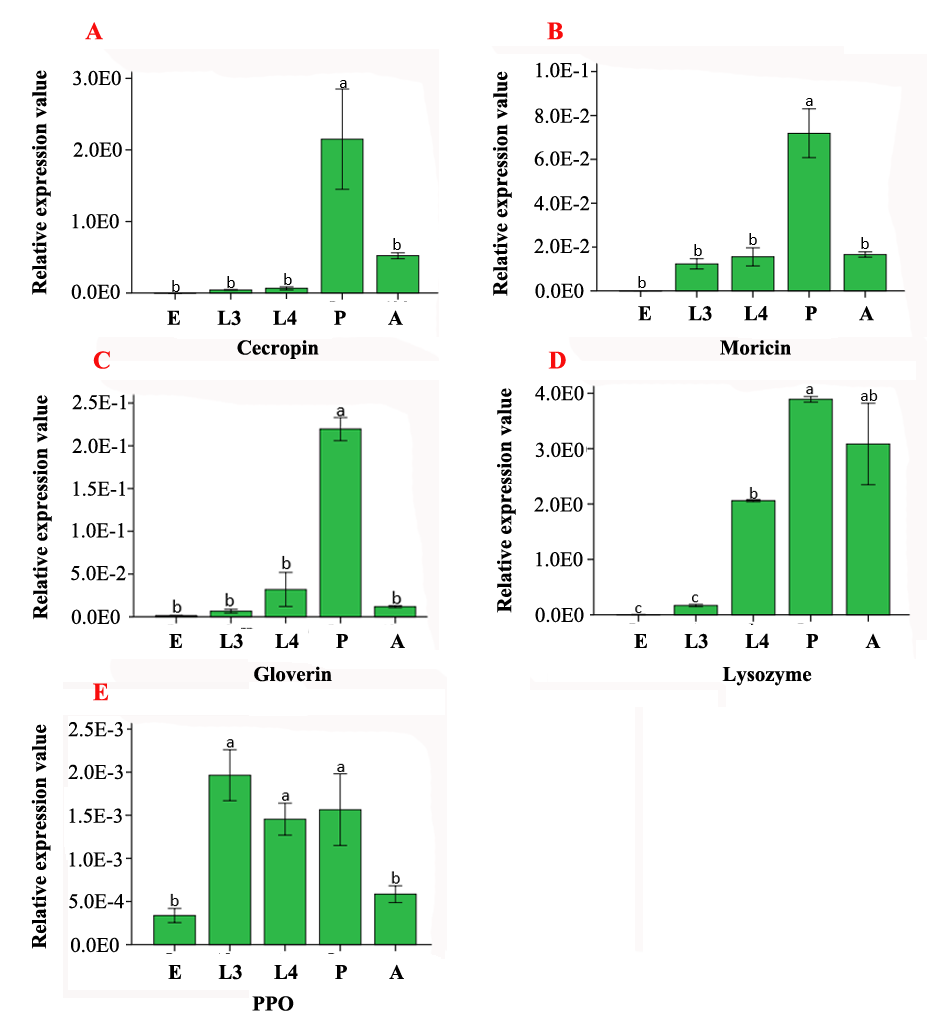


**Fig. S19 Relative expression of immune effector genes at different developmental stages of *P. xylostella* based on qRT-PCR analysis.** Different letters over the columns within a graph denote significant differences (p＜0.05) among different developmental stages of *P. xylostella*, as determined by one-way ANOVA followed by LSD post hoc test. E: Egg; L3: 3^rd^ instar larva; L4: 4^th^ instar larva; P: Pupa; A: Adult.


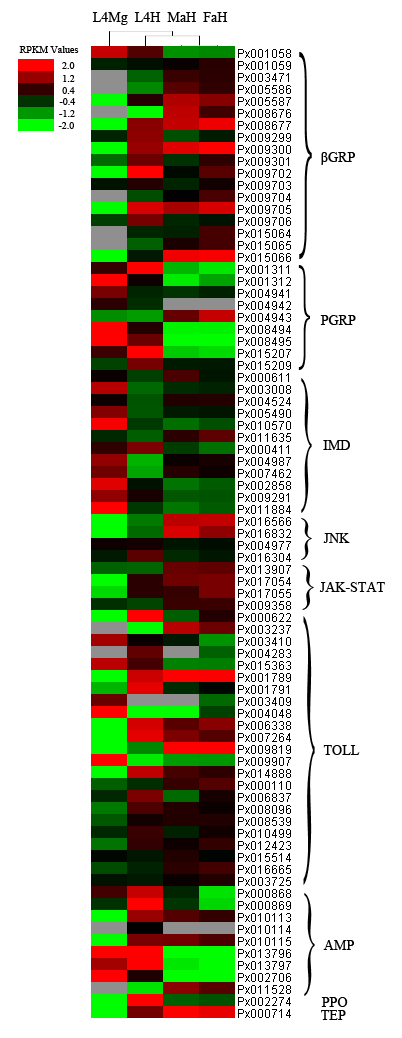


**Fig. S20 Expression profiling of immune genes in the midgut and head of *P. xylostella*.** L4H: 4th instar larva head; L4Mg: 4th instar larval midgut; MaH: Male adult head; FaH: Female adult head. Red represents the up-regulated genes, green indicates the down-regulated genes, and black represents no change in gene expression.


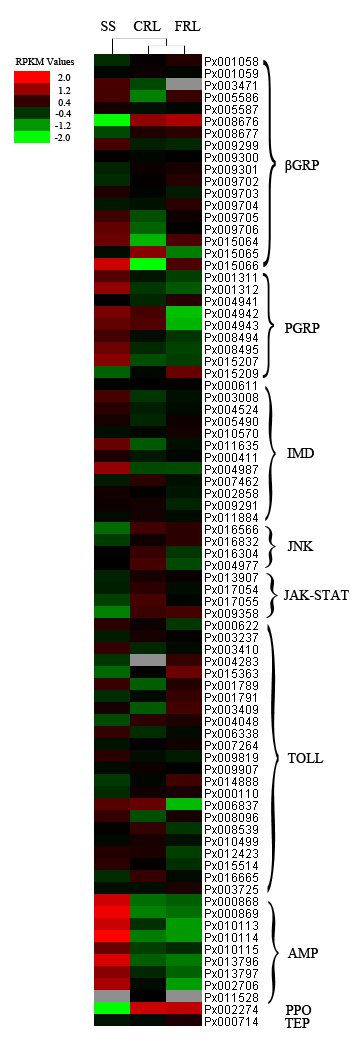


**Fig. S21 Expression profiling of immune genes in the susceptible strain (SS), chlorpyrifos- and fipronil-resistant lines (CRL, FRL) of *P. xylostella*.** Red represents the up-regulated genes; green indicates the down-regulated genes and black represents no change in gene expression.


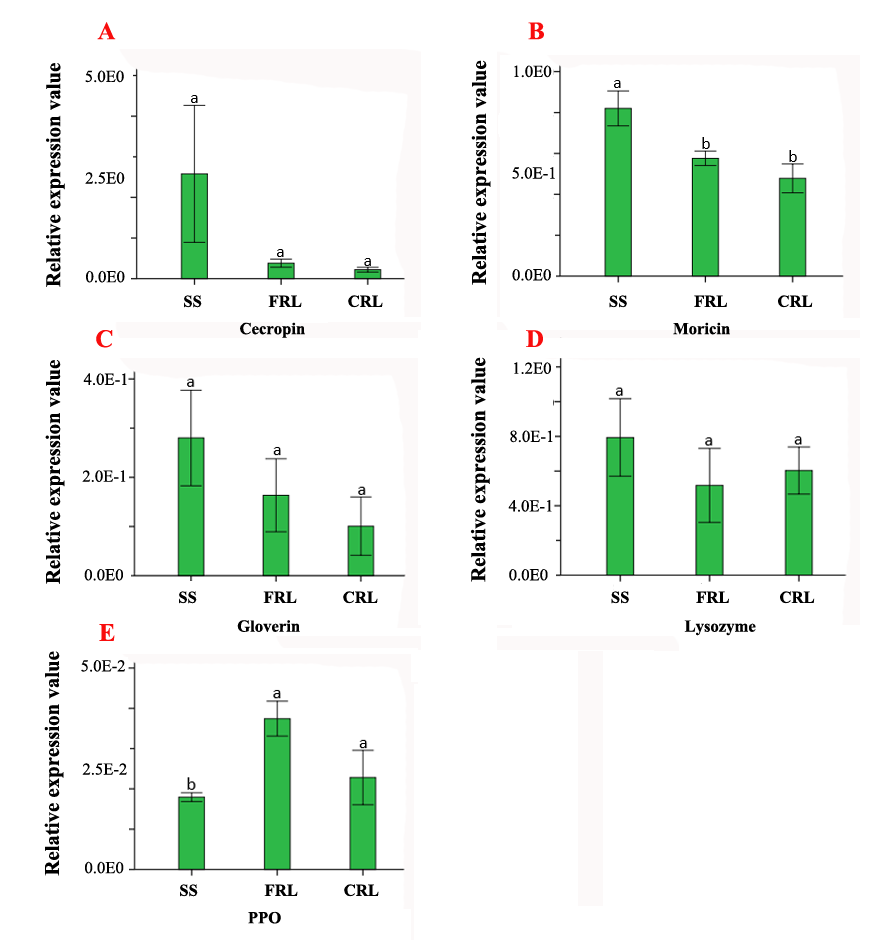


**Fig. S22 Relative expression of immune effector genes in different strains of *P. xylostella* based on qRT-PCR analysis**. Different letters over the columns within a graph denote significant differences (p＜0.05) among different strains of *P. xylostella*, as determined by one-way ANOVA followed by LSD post hoc test. SS: susceptible strain; FRL: fipronil-resistant lines; CRL: chlorpyrifos-resistant lines.
